# Identification of a new aggressive axis driven by ciliogenesis and absence of VDAC1-ΔC in clear cell Renal Cell Carcinoma patients

**DOI:** 10.7150/thno.41001

**Published:** 2020-02-03

**Authors:** Lucilla Fabbri, Maeva Dufies, Sandra Lacas-Gervais, Betty Gardie, Sophie Gad-Lapiteau, Julien Parola, Nicolas Nottet, Monique Meyenberg Cunha de Padua, Julie Contenti, Delphine Borchiellini, Jean-Marc Ferrero, Nathalie Rioux Leclercq, Damien Ambrosetti, Baharia Mograbi, Stéphane Richard, Julien Viotti, Emmanuel Chamorey, Nirvana Sadaghianloo, Matthieu Rouleau, William J. Craigen, Bernard Mari, Stéphan Clavel, Gilles Pagès, Jacques Pouysségur, Frédéric Bost, Nathalie M. Mazure

**Affiliations:** 1 Université Côte d'Azur (UCA), CNRS-UMR 7284-Inserm U1081, IRCAN, Centre Antoine Lacassagne, 33 Ave. de Valombrose, 06189 Nice, France.; 2 Present address: Université Côte d'Azur (UCA), INSERM U1065, C3M, 151 Route de St Antoine de Ginestière, BP2 3194, 06204 Nice Cedex 03, France.; 3 Medical Biology Department, Centre Scientifique de Monaco (CSM), Monaco.; 4 Université Côte d'Azur (UCA), Centre Commun de Microscopie Appliquée, Nice, France.; 5 Institut du thorax, INSERM, CNRS, Univ. Nantes, Nantes, France.; 6 Ecole Pratique des Hautes Etudes, EPHE, PSL Research University, Paris, France.; 7 INSERM UMR 1186, Integrative Tumor Immunology and Genetic Oncology, Gustave Roussy, EPHE, PSL, Fac. de médecine - Univ. Paris-Sud, Université Paris-Saclay, 114 rue Edouard Vaillant, 94800 Villejuif, France.; 8 Centre Antoine Lacassagne, Oncology Department, Nice, France.; 9 Rennes University, Rennes University Hospital, Department of Pathology, Rennes, France.; 10 Centre Hospitalier Universitaire de Nice, Department of Pathology, Nice, France.; 11 REDIR Center, Department of Urology, AP-HP, Bicêtre Hospital, 78 Rue du Général Leclerc, 94270 Le Kremlin-Bicêtre.; 12 Centre Antoine Lacassagne, Statistics Department, Nice, France.; 13 Centre Hospitalier Universitaire de Nice, Department of Vascular Surgery, Nice, France.; 14 Université Côte d'Azur (UCA), CNRS-UMR 7370, LP2M, Nice, France.; 15 Department of Molecular and Human Genetics, The Mitochondrial Diagnostic Laboratory, Baylor College of Medicine, Houston, TX 77030, USA.; 16 Université Côte d'Azur (UCA), CNRS, IPMC, FHUOncoAge, 06560 Valbonne, France.

**Keywords:** Ciliopathy, clear cell Renal Cell Carcinoma, immunotherapy, HIFs, poor prognosis, primary cilium, Voltage-Dependent Anion Channel 1, VDAC1.

## Abstract

**Rationale**: Renal cell carcinoma (RCC) accounts for about 2% of all adult cancers, and clear cell RCC (ccRCC) is the most common RCC histologic subtype. A hallmark of ccRCC is the loss of the primary cilium, a cellular antenna that senses a wide variety of signals. Loss of this key organelle in ccRCC is associated with the loss of the von Hippel-Lindau protein (VHL). However, not all mechanisms of ciliopathy have been clearly elucidated.

**Methods**: By using RCC4 renal cancer cells and patient samples, we examined the regulation of ciliogenesis *via* the presence or absence of the hypoxic form of the voltage-dependent anion channel (VDAC1-ΔC) and its impact on tumor aggressiveness. Three independent cohorts were analyzed. Cohort A was from PREDIR and included 12 patients with hereditary pVHL mutations and 22 sporadic patients presenting tumors with wild-type pVHL or mutated pVHL; Cohort B included tissue samples from 43 patients with non-metastatic ccRCC who had undergone surgery; and Cohort C was composed of 375 non-metastatic ccRCC tumor samples from The Cancer Genome Atlas (TCGA) and was used for validation. The presence of VDAC1-ΔC and legumain was determined by immunoblot. Transcriptional regulation of IFT20/GLI1 expression was evaluated by qPCR. Ciliogenesis was detected using both mouse anti-acetylated α-tubulin and rabbit polyclonal ARL13B antibodies for immunofluorescence.

**Results**: Our study defines, for the first time, a group of ccRCC patients in which the hypoxia-cleaved form of VDAC1 (VDAC1-ΔC) induces resorption of the primary cilium in a Hypoxia-Inducible Factor-1 (HIF-1)-dependent manner. An additional novel group, in which the primary cilium is re-expressed or maintained, lacked VDAC1-ΔC yet maintained glycolysis, a signature of epithelial-mesenchymal transition (EMT) and more aggressive tumor progression, but was independent to VHL. Moreover, these patients were less sensitive to sunitinib, the first-line treatment for ccRCC, but were potentially suitable for immunotherapy, as indicated by the immunophenoscore and the presence of PDL1 expression.

**Conclusion**: This study provides a new way to classify ccRCC patients and proposes potential therapeutic targets linked to metabolism and immunotherapy.

## Introduction

Among the many dysfunctions of tumor cells, the decreased prevalence or loss of the primary cilium, a small sensory organelle in which many signaling factors are known to be concentrated, is being increasingly recognized 1-3. This cilia loss redefines cancer as a form of ciliopathy 4-6. The primary cilium (PC) is a single protrusion emerging from the apical surface of the cell membrane of nearly all mammalian cells during interphase. It senses external signals from the microenvironment and initiates corresponding signaling cascades to the rest of the cell, such as the Hedgehog (Hh) and Wingless (Wnt) pathways 7-11. Its structure is built of a microtubule-based axoneme, which confers mechanical strength and guides the transport of molecules *via* motor-dependent intraflagellar transport (IFT). Any defects in the structure, the activity or the function of the PC affect multiple systems, the consequences of which can be devastating or even life-threatening. There are many phenotypes that are associated with ciliopathies, including renal diseases 12, with the kidneys being among the organs that are most highly affected. A spectrum of renal diseases have been associated with ciliopathic syndromes, including a morphologically heterogeneous group of disorders that have been classified as polycystic kidney disease, renal medullary cystic disease, cystic renal dysplasia and, more recently, renal cell carcinoma 13-15. The von Hippel-Lindau (VHL) protein is encoded by a known tumor suppressor gene, and has been shown to be necessary to maintain cilia 13, 14. Mutations or deletions in the *VHL* gene, in addition to methylation, are characteristic features of: (i) a rare hereditary tumor disease caused by germline alterations of the *VHL* gene 16 and (ii) sporadic clear cell renal cell carcinoma (ccRCC) lacking cilia 17. The VHL protein, a component of an E3 ubiquitin ligase complex, ubiquitylates HIFs and targets them for degradation by the proteasome 18. Interestingly, ccRCCs that are deficient in pVHL cluster into tumors that express either both HIF-1α and -2α or HIF-2α only.

The voltage-dependent anion channel 1 (VDAC1) is the most abundant protein of the mitochondrial outer membrane. VDAC1 has fundamental functions in regulating energy production, calcium signaling and promoting apoptotic signaling 19, 20. A strong relationship between VDAC and hexokinase, the first enzyme of glycolysis, confirms the interconnection between the regulation of glycolysis and mitochondrial respiration. We have further described the role of VDAC1 under hypoxic conditions, in a HIF-1-dependent manner, and showed that a cleaved form of VDAC1 (VDAC1-ΔC) plays a role in promoting resistance to apoptosis, in increasing metabolism and thus in cancer cell survival 21, 22. We characterized its cleavage by the asparagine endopeptidase (Legumain, LGMN) at asparagine 214 to produce VDAC1-ΔC 23. We also showed that the knockout of *Vdac1* in murine embryonic fibroblasts (MEFs) expressing oncogenic RAS potentiates tumor development in mice by promoting metabolic reprogramming, accelerating vascular destabilization and inflammation 23. Finally, a new function for members of the VDAC family has recently been discovered: centrosomal VDAC3 associates with the centrosome *via* Mps1, a protein kinase that plays a role in centriole assembly 24, and this complex leads to aberrant ciliogenesis 24, 25. A similar function has also been described for the centrosomal form of VDAC1. The authors showed that VDAC1 and VDAC3 both negatively modulate PC but with non-redundant functions. However, the mechanisms by which VDACs act on ciliogenesis are unknown.

In the present study, we therefore sought to explore the function of mitochondrial VDAC1-ΔC in the context of ccRCC cells and patients, a rare cancer where ciliopathy and HIF stabilization co-exist. We hypothesized that mitochondrial VDAC1-ΔC could control ciliogenesis. Interestingly, we identified a new group of ccRCC patients in which the primary cilium is re-expressed giving rises to increased tumor aggressiveness. This group is also characterized by the absence of VDAC1-ΔC.

## Materials and Methods

### Cell culture

RCC4/pVHL and 786-O cells were grown in Dulbecco's Modified Eagle's Medium (DMEM) (Gibco-BRL) supplemented with 5% fetal bovine serum with penicillin G (50U/ml) and streptomycin sulfate (50µg/ml). A498 RCC cell lines were purchased from the ATCC (March 3, 2013). The RCC10 cell line was a kind gift from Dr. W.H. Kaelin (Dana-Farber Cancer Institute, Boston, MA). Normal epithelial HK2 cells were a kind gift from Dr I. Rubera (LP2M, Nice, France).

An INVIVO_2_ 200 anaerobic workstation (Ruskinn Technology Biotrace International Plc) set at 1 % oxygen, 94 % nitrogen and 5 % carbon dioxide was used for hypoxic conditions.

### Pharmacological inhibitors and chemicals

3BP was from Sigma. Sunitinib was from Centre Antoine Lacassagne.

### RNA interference

The 21-nucleotide RNAs were chemically synthesized (Eurogentec, Seraing, Belgium) and previously described. The siRNA sequences, all validated, were as follows: siCtl (forward) 5'-CCU-ACA-UCC-CGA-UCG-AUG-AUG-TT-3' 26, siVDAC1 (forward) 5'-GAUACACUCAGACUCUAAA -3' 22, 27, siHIF-1α (forward) 5'- CUG-AUG-ACC-AGC-AAC-UUG-ATT-3' 22, 26, 28, siHIF-2α (forward) 5'- CAG-CAU-CUU-UGA-UAG-CAG-UTT-3' 26. siLGMN also known as AEP has been described previously 27. siIFT20 and siGLI1 were from Mission esiRNA Sigma, and were a heterogenous mixture of siRNAs that all target the same mRNA sequence.

### Quantitative real-time PCR analysis

For tumor samples, total RNA was extracted with the RNeasy FFPE Kit (QIAGEN, Hilden, Germany). For cells, total RNA was extracted with the RNeasy Mini Kit (QIAGEN, Hilden, Germany). The amount of RNA was evaluated with a NanoDrop™ spectrophotometer (ThermoFisher Scientific, Waltham, MA USA). One µg of total RNA was used for reverse transcription, using the QuantiTect Reverse Transcription kit (QIAGEN, Hilden, Germany), with a blend of oligo (dT) and random primers to prime first-strand synthesis. SYBR master mix plus (Eurogentec, Liege, Belgium) and specific oligonucleotides (Sigma Aldrich) were used for qPCR. Primer sequences used are: *GLI1* (forward: 5'-TGCAGTAAAGCCTTCAGCAATG -3'; reverse: 5'-TTTTCGCAGCGAGCTAGGAT- 3'), *IFT20* (forward: 5'-GGTATCGGGTTGAATATGAAG-3'; reverse: 5'-GACATAGGTCATTGGTCAAG-3'). *LGMN* (forward: 5'-ACTATGATGAGAAGAGGTCC-3'; reverse: 5'-GGTGGAGATTGTTTTGTTTC3'); *PDL1* (forward: 5'-ATGCCCCATACAACAAAATC-3'; reverse: 5'-GACATGTCAGTTCATGTTCAG3'); *STAT3* primers was a kind gift from Dr J. Gilleron (C3M, Nice).

### Immunoblotting

Cells were lysed in 1.5x Laemmli buffer and the protein concentration determined using the BCA assay. 40 µg of protein from whole cell extracts was resolved by SDS-PAGE and transferred onto a PVDF membrane (Millipore). Membranes were blocked in 5% non-fat milk in TN buffer (50 mM Tris-HCl pH 7.4, 150 mM NaCl) and incubated in the presence of the primary and then secondary antibodies in 5% non-fat milk in TN buffer. The rabbit polyclonal antibody to central regions of VDAC1 was purchased from Abcam (ab15895). Rabbit polyclonal anti-HIF-1α antibody (antiserum 2087) was produced and characterized in our laboratory 29. The antibodies against HIF-2α (NB100-122) and ARL13b (NBP2-15463) were purchased from Novus Biologicals (Littleton, CA). Mouse anti-acetylated α-tubulin (T7451), anti-β-tubulin, HSP90 and β-actin were from Sigma. ECL signals were normalized to either β-tubulin or HSP90. Anti-LGMN antibody (AF2058) was from R&D system. After washing in TN buffer containing 1 % Triton-X100 and then in TN buffer, immunoreactive bands were visualized with the ECL system (Amersham Biosciences).

### Immunocytochemistry

Cells were fixed in 3% paraformaldehyde and permeabilized with Triton X-100. Primary antibodies included mouse anti-acetylated α-tubulin (Sigma-Aldrich, Basel, Switzerland; 1:400 dilution); rabbit anti-Arl13b (Novusbio, Abingdon, United Kingdom; 1:400 dilution). Alexa Fluor 594- and 488-conjugated secondary goat anti-mouse or goat anti-rabbit antibodies (Molecular Probes, Carlsbad, CA) were used at 1:400. Cells were visualized by wide-field, fluorescence microscopy using a DM5500B upright stand (Leica, Germany) with a 40X oil objective NA 1.00. The cubes used were A4 (excitation filter BP 360/40, dichroic mirror 400, emission filter BP 470/40), L5 (BP 480/40, 505, BP 527/30), and TX2 (BP 560/40, 595, BP645/75). Acquisitions were done with an Orca-ER camera (Hamamatsu, Japan). Cells were also visualized using the confocal microscope, Axiovert 200M inverted stand (Zeiss, Germany). Objectives 10X dry NA 0.3 and/or 25X multi immersion (oil, glycerol, water) NA 0.75, and/or 40X oil 1.3 NA and/or 63X oil 1.4 NA were used. The LASERs used were diode 405 nm, and/or Argon 488 nm, and/or HeNe 543 nm. The microscope was equipped with an automated xy stage for mosaic acquisitions.

Cilia frequency was counted manually from scans using a 40X digital zoom for 100-300 nuclei.

### Immunohistochemistry

Normal and renal cell carcinoma tissue sections (5 µm) were obtained from the Department of Pathology of Centre Hospitalier Universitaire de Nice, Nice (France). The sections had been formol fixed within 1h of surgery, for a total of 72h before being paraffin-embedded. After dewaxing, rehydrating, antigen retrieval was achieved by boiling in 0.01 M citrate buffer for 20 min. For immunofluorescence detection of primary cilia, sections were incubated with mouse anti-acetylated α-tubulin (Sigma-Aldrich, Basel, Switzerland; 1:50 dilution), rabbit anti-Arl13b (Novusbio, Abingdon, United Kingdom; 1:50 dilution) or rabbit anti-PDL1 (eBioscience ThermoFisher Scientific, Waltham, USA; 1:10 dilution) primary antibodies, then followed by goat anti-mouse secondary antibodies conjugated to Alexa 594 and goat anti-rabbit secondary antibodies conjugated to Alexa 488 (Molecular Probes, Invitrogen, Basel, Switzerland; 1:100 dilution). Nuclei were labeled with 2 mg/mL 4-6-diamidino-2-phenylindole (DAPI). Cilium counting was performed by focusing up and down on the microscope to capture cilia and nuclei that lay in different focal planes within the section. Images were obtained using an Axiovert 200M inverted stand (Zeiss, Germany) with a 40X oil objective 1.3 NA with samples mounted in an immersion medium (water). A diode 405 nm, Argon 488 nm and HeNe 543 nm laser was used. Optical sections were 0.3 µm thick and stacks were made encompassing a Z-plane depth of 0.5 µm. The number of cilia was counted manually from scans using a 40X digital zoom for at least 500 nuclei.

### FACS analysis

For determination of IFT20 expression in RCC4+pVHL-, RCC4-, RCC4 siCtl-, RCC4 siVDAC1-, RCC4 siLGMN- cells, cellular suspensions (1 × 10^6^ cells) were resuspended in 4% PFA for 15 min and washed 3 times in cold PBS/BSA 0.1%. Cell suspensions were then incubated with a polyclonal rabbit anti-IFT20 (1 µg/1 × 10^6^ cells; Proteintech) for 1 h at 4°C in PBS/BSA 0.1% buffer. After washing, cells were incubated with Alexa 488-conjugated secondary goat anti-rabbit antibody (µg/1 × 10^6^ cells;) for 30 min and washed prior to analysis. Samples were collected with Miltenyi MCSQuant10 (Bergisch Gladbach, Germany) and analyzed with FlowJo Software. Secondary antibody in the control experiment was identical to that described *supra*.

### Invasion assay

The invasion assay was performed using cell culture inserts with 8.0 µm pore transparent PET membrane coated with 10 μg/mL fibronectin. Inserts were coated with 2 μg/μL of Matrigel and incubated for 3 h at 37 °C in a CO_2_ incubator. Briefly, overnight serum-starved cells (8×10^4^ cells) were seeded into the top chamber in medium without FBS, while medium with 10% FBS was present in the bottom chamber. The cells were incubated for 24 h. Media and remaining cells were removed from the top chamber with a cotton swab and washed twice with PBS. The bottom chamber was aspirated and washed twice with PBS. Inserts were fixed with 4% PFA. Invasiveness was assayed in triplicate for each condition, in at least three independent experiments. Cells that invaded the Matrigel and migrated through the filter and adhered to the lower surface were stained for 10 min with 0.5% crystal violet in 25% methanol. Inserts were rinsed in distilled water until no additional stain leached and were air-dried overnight. Crystal violet was extracted from the invading cells by adding 600 µL of 0.1 M sodium citrate in 10% acetic acid. Absorbance was measured spectrophotometrically at 585 nm using the Spectronic GENESYS 5 (Milton Roy, Rochester, NY).

### Tumor spheroid invasion assay

A cell suspension of 15 × 10^3^ cells/mL was prepared and 200 µL aliquots were put in 24 wells containing solidified agarose 1% and incubated at 37^o^C for 48h. Spheroids containing 3000 cells each were placed on the inside of the cover of a culture dish and incubated at 37^o^C. Formed spheroids were transferred into wells containing matrigel 25% + 1 mg/mL 3D collagen I gel and left to grow for the designated time (24h, 48h and 72h). Diameters of the spheroids were monitored by an Evos optical microscope. Cell invasion through the surrounding collagen was measured using the ImageJ software and the final spheroid size was compared to the initial size at time zero. At least 8 spheroids were analyzed per condition and at least three independent experiments were performed.

### CAM assay

Fertilized chicken eggs (*Gallus gallus*) (EARL Morizeau, Dangers, France) were handled as previously described 30. On embryonic day 9, a plastic ring was placed on the CAM, and 1 million RCC4 or RCC4+pVHL cells in 20 µL of medium were deposited on the surface. Digital photos were taken under a stereomicroscope.

### Wound-healing assay

The equivalent number of RCC4 and RCC4+pVHL cells were seeded and incubated for 24 h. Cells were photographed at the time of insert removal (0 h), then 8 h, 16 h and 20 h after. The percentage of the scratched area at each time point was calculated with ImageJ.

### Immunophenoscore

Immunophenoscore is a predicator of the response to checkpoint blockade established by Charoentong et al. based on a panel of immune genes for classification of patients likely to respond to therapy with antibodies targeting CTLA-4 and PD-1 with superior performance 31.

### Patients and cohorts

RCC was classified according to the tumor, node and metastasis (TNM) system developed by the American Joint Committee on Cancer (AJCC). RCC was classified from Stage I to Stage IV according to the TN and a prognostic score.

#### Stages

- stage I: T1 N0 M0
- stage II: T2 N0 M0
- stage III: T3 or N1 with M0
- stage IV: T4 or M1

#### Patients from Nice (Table S1)

Tissue samples from 19 patients with ccRCC who had undergone surgery in the Urology and Pathology Departments of the Nice University Hospital were selected. For each patient, a piece of fresh tumor was embedded in paraffin (IF) and a piece was immediately frozen (immunoblot). For each patient, tumor diagnosis was based on pathology and on cytogenetic analyses, as defined by the 2016 World Health Organization criteria. This prospective study was approved by the institutional review board and was conducted in accordance with the Declaration of Helsinki.

#### Cohort A (PREDIR; Table S2)

A series of 32 renal tumors were obtained thanks to the PREDIR Center (French Kidney Cancer Consortium coordinated by S. Richard) composed of 12 VHL tumor-associated and 22 sporadic RCC that were verified as clear-cell renal cell carcinomas. Part of the microarray transcriptome analysis of this series has been previously reported 32.

#### Cohort B (Table S3)

Tissue samples from 43 patients with ccRCC who had undergone surgery in the Urology Department of the Rennes University Hospital were selected. As defined by the 2016 World Health Organization criteria, diagnosis was based on the pathology and on cytogenetic analyses. This retrospective study was approved by the institutional review board and was conducted in accordance with the Declaration of Helsinki.

#### Cohort C (TCGA) has been previously described 33

### Gene expression microarray analysis

Normalized RNA sequencing (RNA-Seq) data produced by The Cancer Genome Atlas (TCGA) were downloaded from cbioportal (www.cbioportal.org, TCGA Provisional; RNA-Seq V2). Different parameters were available for 375 ccRCC tumor samples, with information for VHL status (methylation, mutation and deletion) 34, 35. We performed a differential expression analysis between patients with a “primary cilium” signature and patients with a “no primary cilium” signature using the Bioconductor package DESeq2.

The results published here are in whole or in part based upon data generated by the TCGA Research Network: http://cancergenome.nih.gov/.

### Database analysis

To assess the effect of the presence or absence of the primary cilium in ccRCC from Cohort C, we performed a differential analysis between the group expressing the primary cilium (n=48 patients) and the group expressing no primary cilium (n=327 patients) by computing the ratio and p-values obtained with a Wilcoxon rank sum test. We then performed a functional and pathway enrichment analysis on differentially expressed genes (p-value < 0.05 and absolute ratio > 0.7) based on Reactome databases using the geneSCF tool 36. The terms are considered significant only if enriched with a p-value < 0.05.

### Statistics

All values are the means±SEM. Statistical analyses were performed using the Student's *t* test in Microsoft Excel. The *p* values are indicated. All categorical data used numbers and percentages. Quantitative data were presented using the median and range or mean. Differences between groups were evaluated using the chi square test for categorical variables and the Student's *t* test for continuous variables. Analyses were performed using SPSS 16.0 statistical software (SPSS Inc., Chicago, Ill). All statistical tests were two-sided, and *p*-values <0.05 indicated statistical significance whereas *p*-values between 0.05 and 0.10 indicated a statistical tendency.

### Statistics for patients

The Student's* t*-test was used to compare continuous variables and chi-square test, or Fisher's exact test (when the conditions for use of the *χ*^2^-test were not fulfilled), were used for categorical variables. To guarantee the independence of the primary cilium as a prognostic factor, the multivariate analysis was performed using Cox regression adjusted to the stage and age. DFS was defined as the time from surgery to the appearance of metastasis. OS was defined as the time between surgery and the date of death from any cause, censoring those alive at last follow-up. The Kaplan-Meier method was used to produce survival curves and analyses of censored data were performed using Cox models. All analyses were performed using R software, version 3.2.2 (Vienna, Austria, https://www.r-project.org/).

## Results

### Low VDAC1 and LGMN expression levels are linked to poor prognosis in ccRCC patients

**Figure 1:**
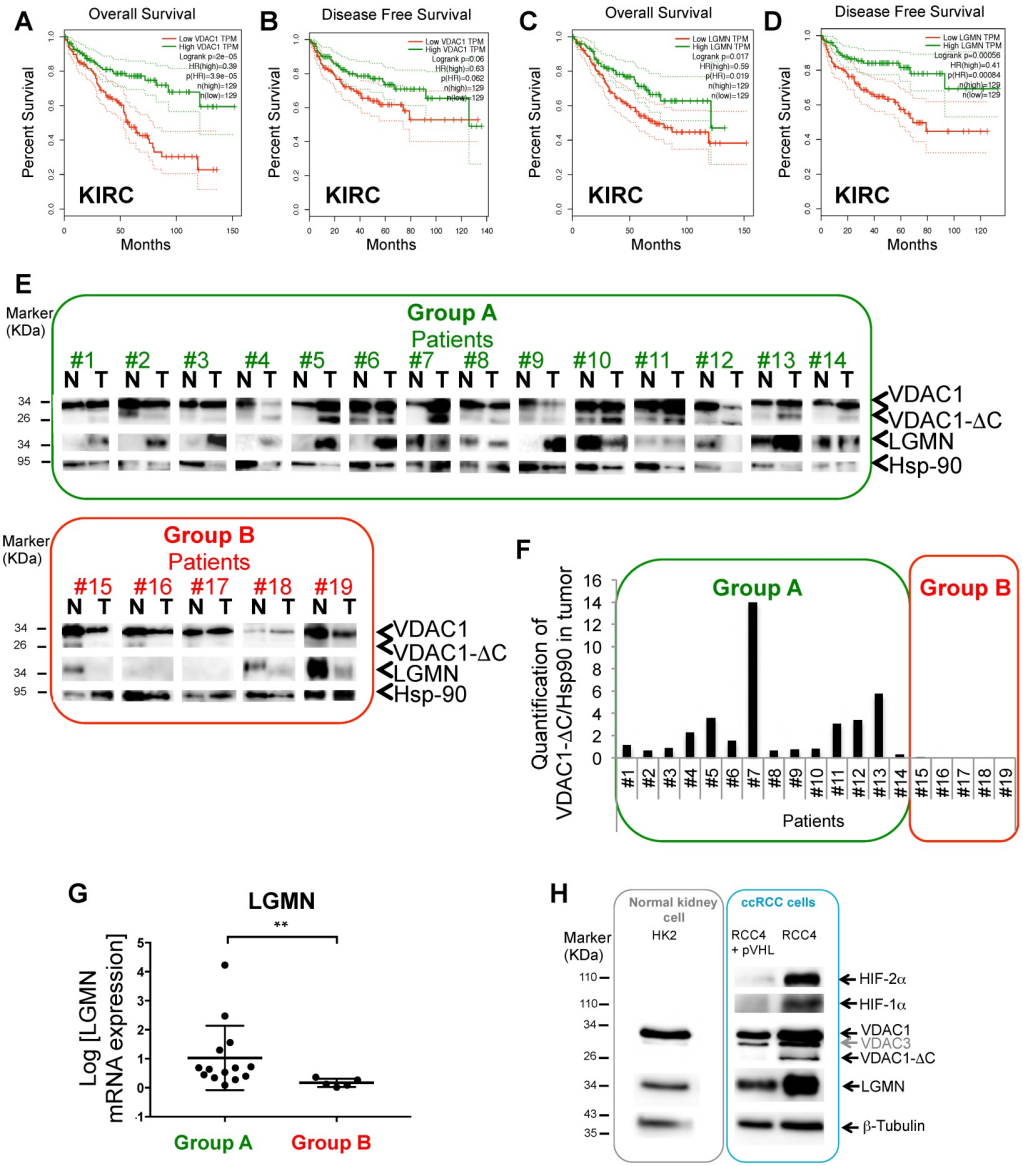
** A and B,** Overall survival (**A**) and Disease free survival (**B**) for VDAC1 expression was calculated from Kidney renal clear cell carcinoma (KIRC) (Gepia.cancer-pku.cn) using the third quartile for the cutoff. **C and D**, Overall survival (**C**) and Disease free survival (**D**) for LGMN expression was calculated from KIRC. **E**, Tissues lysates (Normal (N), Tumoral (T)) of 19 patients were analyzed by immunoblotting for VDCA1 and LGMN. Hsp-90 was used as a loading control. Group A: high level of VDAC1 + VDAC1-ΔC and LGMN in T. Group B: low level of VDAC1 and absence of VDAC1-ΔC and LGMN. **F**, Expression of VDAC1-ΔC and Hsp-90 proteins was quantified in tumoral tissues (T) and VDAC1-ΔC/Hsp-90 ratio was obtained in each patient. **G**, Graphic representation of LGMN mRNA expression in patients from Group A compared to patients from Group B. **H**, HK2, RCC4+pVHL, RCC4, cell lysates were analyzed by immunoblotting for HIF-2α, HIF-1α, LGMN and VDAC1. β-tubulin and Hsp-90 were used as a loading control.

By interacting with hexokinase, or members of the Bcl-2 family, VDAC1 supports glycolysis and apoptosis is prevented. VDAC is thus involved in determining cellular survival or death, which is particularly relevant to cancer cells. To explore its possible role in ccRCC (kidney renal clear cell carcinoma, KIRC), we first interrogated the Gene Expression Profiling Interactive Analysis (GEPIA) human dataset. Interestingly, the patients' overall survival (OS; Figure 1A) and disease-free survival (DFS; Figure 1B) plots revealed a direct correlation between low levels of VDAC1 and a poor prognosis. As we had previously shown a link between the asparagine endopeptidase (LGMN) and VDAC1 in hypoxia (Supplementary Fig. S1A) 27, 37, we also explored the expression level of LGMN in the same cohort of ccRCC patients. Similar to VDAC1, both OS (Figure 1C) and DFS (Figure 1D) showed a strong correlation between low levels of LGMN and a poor prognosis. We obtained 19 tissue samples of ccRCC patients from the Pathology Department of Nice (CHU; Table S1). Fourteen out of 19 (73.7%) were classified with high VDAC1 expression and the presence of VDAC1-ΔC in tumor tissues (defined as group A) whereas five out of 19 (26.3%) were classified with a low level of VDAC1 and the absence of VDAC1-ΔC (defined as group B; Figure 1E-F). In group A, VDAC1-ΔC and LGMN were present in tumor (T) tissues, except for patients #7 and #12 (Supplementary Fig. S1B). In contrast, group B showed no VDAC1-ΔC in tumor (T) tissues in parallel with weak or absent expression of LGMN. Moreover, LGMN protein expression (Figure 1E) also correlated to its mRNA level (Figure 1G). Of the 19 tissue samples from the ccRCC patients, HIF-2 and sometimes HIF-1 was present in each group suggesting that this classification does not depend on the HIF status (Figure S1C). VDAC1-ΔC expression was also analyzed in ccRCC cell lines. HK2, kidney epithelial cells from normal kidney, did not express VDAC1-ΔC under normoxic conditions (Figure 1H). As previously shown 38, analysis of *VHL* mutant RCC4 cell lines, in which the wild-type *VHL* gene has been restored (RCC4+pVHL), and thus mimicking normoxia with no stabilization of either HIF-1α or -2α, showed no VDAC1-ΔC whereas RCC4 cells with both stabilization of HIF-1α or -2α expressed VDAC1-ΔC. Moreover, LGMN was expressed in each cell line with higher expression in cells defective for pVHL (Figure 1H).

These results suggest a strong link between VDAC1/VDAC1-ΔC and LGMN in the ccRCC context and describe two groups of ccRCC patients with distinct prognoses.

### The presence of VDAC1-ΔC in RCC4 and 786-O cells decreases or abolishes ciliation

**Figure 2:**
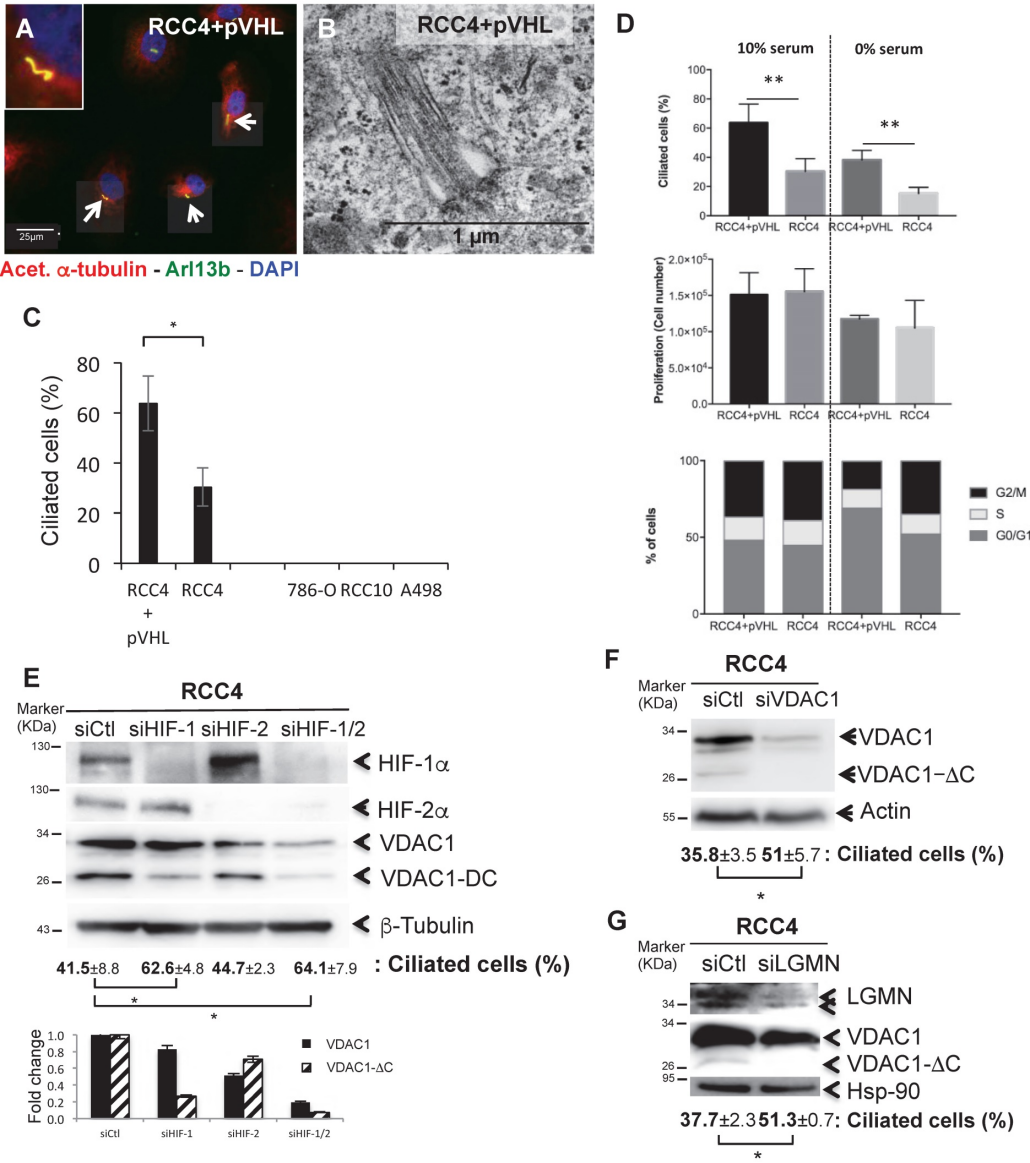
**The presence of VDAC1-DC in ccRCC cells decreases or abolishes ciliation. A**, Triple immunofluorescence labeling and merged images with acetylated α-tubulin (Acet. α-tubulin in red), Arl13b (in green) and DAPI (in blue). **B**, Electron microscopy of RCC4+pVHL cells. **C**, Quantitative analysis of the ciliation percentage was assessed by confocal fluorescence microscopy (n=100-300 cells). **D**, Both cell lines were seeded at the same density and incubated in Nx for 48h with or without serum. Percentage of ciliated cells, proliferation and FACS analysis were measured. The mean ± SEM is representative of three independent experiments carried out in duplicate. **E, F and G**, RCC4 cells were transfected with control siRNA (siCtl), (**E**) siHIF-1α, siHIF-2α and siHIF-1/2α, (**F**) siVDAC1 and (**G**) siLGMN. Cell lysates were analyzed by immunoblotting for HIF-1α, HIF-2α, VDAC1, LGMN and β- tubulin/Actin or HSP90 were used as a loading control. Quantitative analysis of the ciliation percentage was assessed by confocal fluorescence microscopy (n=100-300 cells). A * p<0.05 shows significant differences. Quantification of VDAC1 and VDAC1-ΔC protein levels (E). Experiments have been proceeded without serum.

As ccRCC is associated with the loss of VHL function, deregulation of the hypoxia pathway and the loss of primary cilia 39, we investigated a potential role of VDAC1 in ciliogenesis. Using mouse anti-acetylated α-tubulin (which are microtubule proteins that are enriched in the axonemes of most primary cilium), rabbit polyclonal ARL13B antibodies counterstained with DAPI for immunofluorescence (Figure 2A), and electron microscopy (Figure 2B) we found the presence of primary cilia in RCC4+pVHL and RCC4 cells only. A high proportion of ciliated cells were observed in RCC4+pVHL and RCC4 cells (between 60% to 32%), whereas no primary cilia were detected in 786-O, RCC10 and A498 (Figure 2C). RCC4 had 50% fewer primary cilia compared to RCC4+pVHL in similar conditions of proliferation. Cell proliferation was tested as the primary cilium is highly dependent on the cell cycle. RCC4 and RCC4+pVHL were cultured in the presence or absence of serum where cell growth was arrested (Figure 2D). No significant difference was observed between the two cell types. Following experiments were performed in the absence of serum in order to magnify the number of ciliated cells. However, as RCC4+pVHL and RCC4 cells presented a lower percentage of ciliated cells, these results suggest that ciliogenesis and cell cycle could be deregulated and uncoupled in these tumoral cells. The knockdown of HIF-1α alone or both HIF-1α and -2α using specific siRNAs, decreased the expression of VDAC1-ΔC and concomitantly increased the percentage of ciliated cells compared to siCtl (Figure 2E). However, the knockdown of HIF-2α alone had no effect on VDAC1-ΔC and the primary cilium. Finally, siRNA targeting VDAC1 in the RCC4 cell line statistically increased the percentage of ciliated cells by more than 1.4-fold (Figure 2F). In order to block the hypoxic cleavage of VDAC1, LGMN was silenced in RCC4 cells (Figure 2G). VDAC1-ΔC totally disappeared and cells expressed a higher percentage of primary cilia, which was similar to what we have observed by downregulating VDAC1 expression.

These results strongly suggested that VDAC1-ΔC controls resorption of the primary cilium in the HIF-1/LGMN-dependent model of ccRCC.

### The GLI1/IFT20 signature correlates to the primary cilium and VDAC1

**Figure 3:**
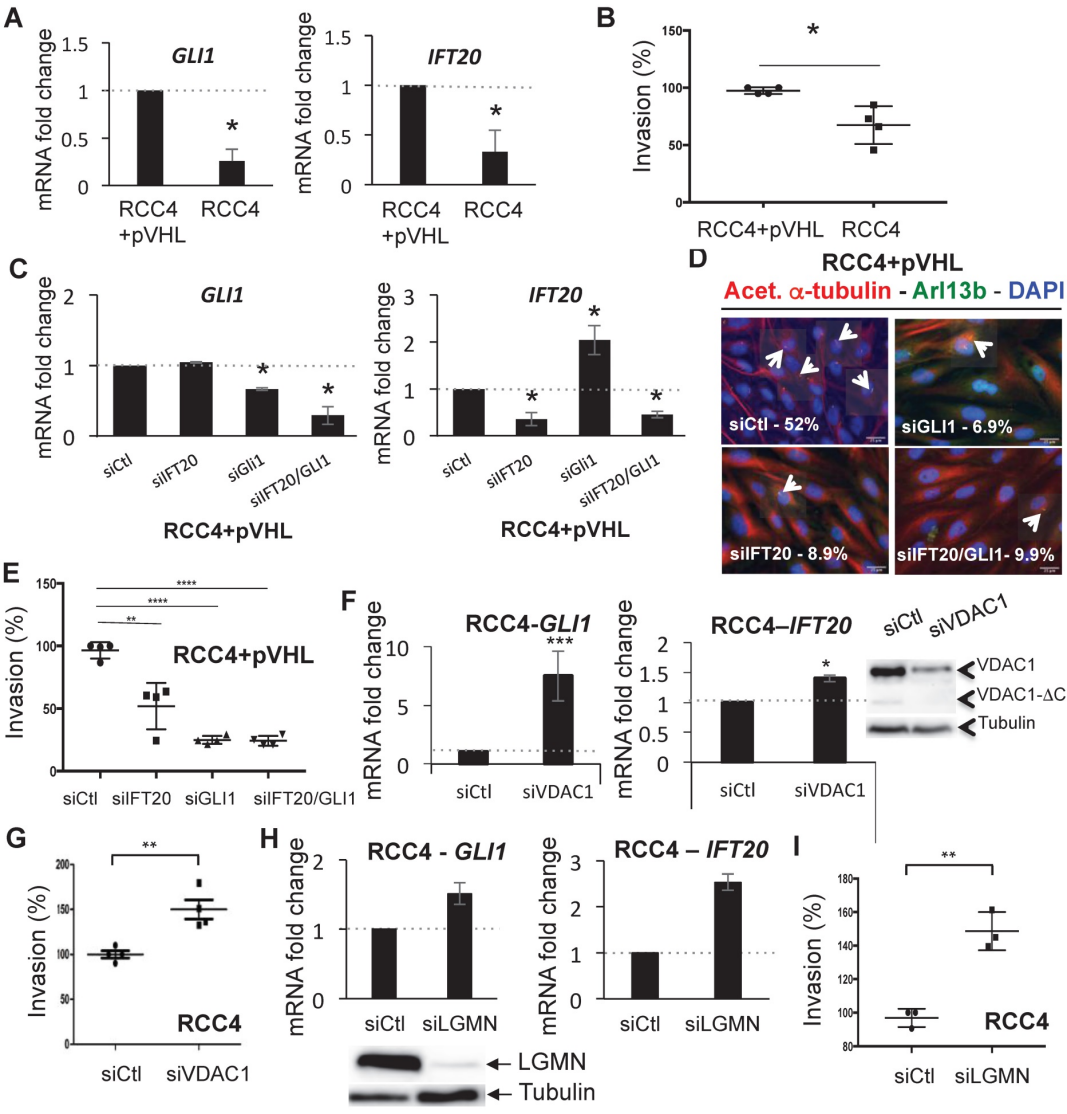
** GLI1/IFT20 signature is correlated to primary cilium and VDAC1. A**, Histograms represent the expression of the mRNA of *GLI1* (left panel) and *IFT20* (right panel) in RCC4 cells compared to RCC4+pVHL in Nx. **B**, Graphic representation of the Boyden chamber cell-based invasion assay using RCC4+pVHL and RCC4 cells. **C**, RCC4+pVHL cells were transfected with control siRNA (siCtl), si*GLI1* (40nM), si*IFT20* (40nM) and si*IFT20/GLI1* (40nM+40nM). Quantitative analysis of the ciliation percentage in RCC4+pVHL cells was assessed by confocal fluorescence microscopy (n=100-300 cells). **D**, Triple immunofluorescence labeling and merged images with acetylated a-tubulin (Acet. a-tubulin in red), Arl13b (in green) and DAPI (in blue). **E**, Graphic representation of the Boyden chamber cell-based invasion assay using RCC4+pVHL cells transfected with control siRNA (siCtl), si*GLI1* (40nM), si*IFT20* (40nM) and si*IFT20/GLI1* 40nM+40nM). **F,** Histograms represent the expression of the mRNA of *GLI1* (left panel) and *IFT20* (right panel) in RCC4 cells transfected with siRNA VDAC1. Cell lysates from the same experimetn were analyzed by immunoblotting. **G,** Graphic representation of the Boyden chamber cell-based invasion assay using RCC4 cells transfected with siRNA VDAC1 compared to Ctl (siCtl). **H**, Histograms represent the expression of the mRNA of *GLI1* (left panel) and *IFT20* (right panel) in RCC4 cells transfected with siRNA LGMN. Cell lysates from the same experimetn were analyzed by immunoblotting. **I**, Graphic representation of the Boyden chamber cell-based invasion assay using RCC4 cells transfected with siRNA LGMN compared to Ctl (siCtl). The mean ±SEM is representative of three independent experiments. A * p<0.05, ** p<0.005 and *** p<0.0005 show significant differences. Experiments have been proceeded without serum.

To further reinforce the link between VDAC1-ΔC and the percentage of ciliated cells, we evaluated the expression of genes involved in the biogenesis and the activity of the primary cilium. The analysis of our transcriptomic data in WT MEF in hypoxia *versus* normoxia 23 highlighted differences in the expression of genes related to the primary cilium. Given that mRNA levels of the *GLI1* transcription factor and the intraflagellar transport protein 20 (*IFT20*) had been shown to be modified in hypoxia, -0.59 and +0.91 respectively (23, NCBI Gene Expression Omnibus (GEO) (http://www.ncbi.nlm.nih.gov/geo/) under the series record number GSE63247) and because this combination, among all those tested, proved to be a fair reflect of the presence or absence of the primary cilium, we chose to examine these two genes as indicators for the activity and formation of the primary cilium. The less ciliated RCC4 cells had low expression levels of both *GLI1* and *IFT20* and could be classified as *GLI1*-/*IFT20*- cells in comparison to the more ciliated RCC4+pVHL cells (Figure 3A). Similarly, IFT20 protein abundance was decreased in RCC4 compared to RCC4+pVHL cells demonstrating a clear correlation between the mRNA and protein levels of IFT20 (Figure S2A). Moreover, a cell invasion assay to evaluate aggressiveness demonstrated that RCC4 cells were less invasive compared to RCC4+pVHL cells (Figure 3B). This result was also confirmed using the three-dimensional, *in vitro*, chicken chorioallantoic membrane (CAM) assay and a migration assay (Figure S3).

Moreover, the RCC4+pVHL cells presented an epithelial-mesenchymal transition signature with higher expression of *TWIST*, *SNAIL* and *SLUG* compared to RCC4 cells reinforcing the aggressivity profile often associated with EMT with a concomitant increase in glycolytic capacity. We then knocked down *GLI1* and *IFT20* in RCC4+pVHL cells (Figure 3C), and observed no change in the expression of VDAC1 or VDAC1-ΔC (Figure S2B) but a decrease in the presence of primary cilia (Figure 3D) associated with decreased invasion (Figure 3E). Moreover, knocking down *GLI1* and *IFT20* in RCC4 cells (Figure S2C) also increased expression of VDAC1 and, subsequently, increased expression of VDAC1-ΔC (Figure S2D). In contrast, RCC4 cells transfected with siRNA to VDAC1 presented a gene expression profile of *GLI1*+ and *IFT20*+ cells and overexpression of IFT20 compared to siRNA to Ctl (Figure 3F and Figure S2E). Moreover, in these cells, downregulation of VDAC1 was sufficient to increase the invasive potential (Figure 3G). RCC4 cells transfected with siRNA targeting *LGMN* presented a *GLI1*+/*IFT20*+ signature and overexpression of IFT20 (Figure 3H and Figure S2E) and were more aggressive (Figure 3I), which was similar to what we observed by downregulating VDAC1. To confirm the impact of the primary cilium on invasion, we silenced an independent marker of the primary cilium structure, *KIF3A*. RCC4+pVHL cells transfected with siRNA to KIF3A showed a significant decrease in ciliated cells, which was linked to a decrease in invasion (Figure S2F). In parallel, we overexpressed GLI1 and IFT20 in RCC4 cells (Figure S4A), and observed a reproducible increase in the percentage of ciliated cells (5%) (Figure S4B) associated with increased invasion (Figure S4C). Moreover, overexpression of LGMN in RCC4+pVHL cells (Figure S4D) led to a GLI1- and IFT20+ gene expression profile (Figure S4E) correlated with a decreased percentage of ciliated cells (Figure S4F) and decreased invasion potential (Figure S4G).

These results demonstrated that downregulation of *GLI1* and/or *IFT20* expression correlated with the decrease or absence of primary cilia expression in ccRCC cells with VDAC1-ΔC expression and *vice versa*. Moreover, the decrease in VDAC1/VDAC1-ΔC expression in RCC4 cells increased the percentage of ciliated cells and was correlated with increased invasive potential.

### The 2-gene signature is predictive of the presence of both the VDAC1-ΔC and the primary cilium in ccRCC patients

**Figure 4:**
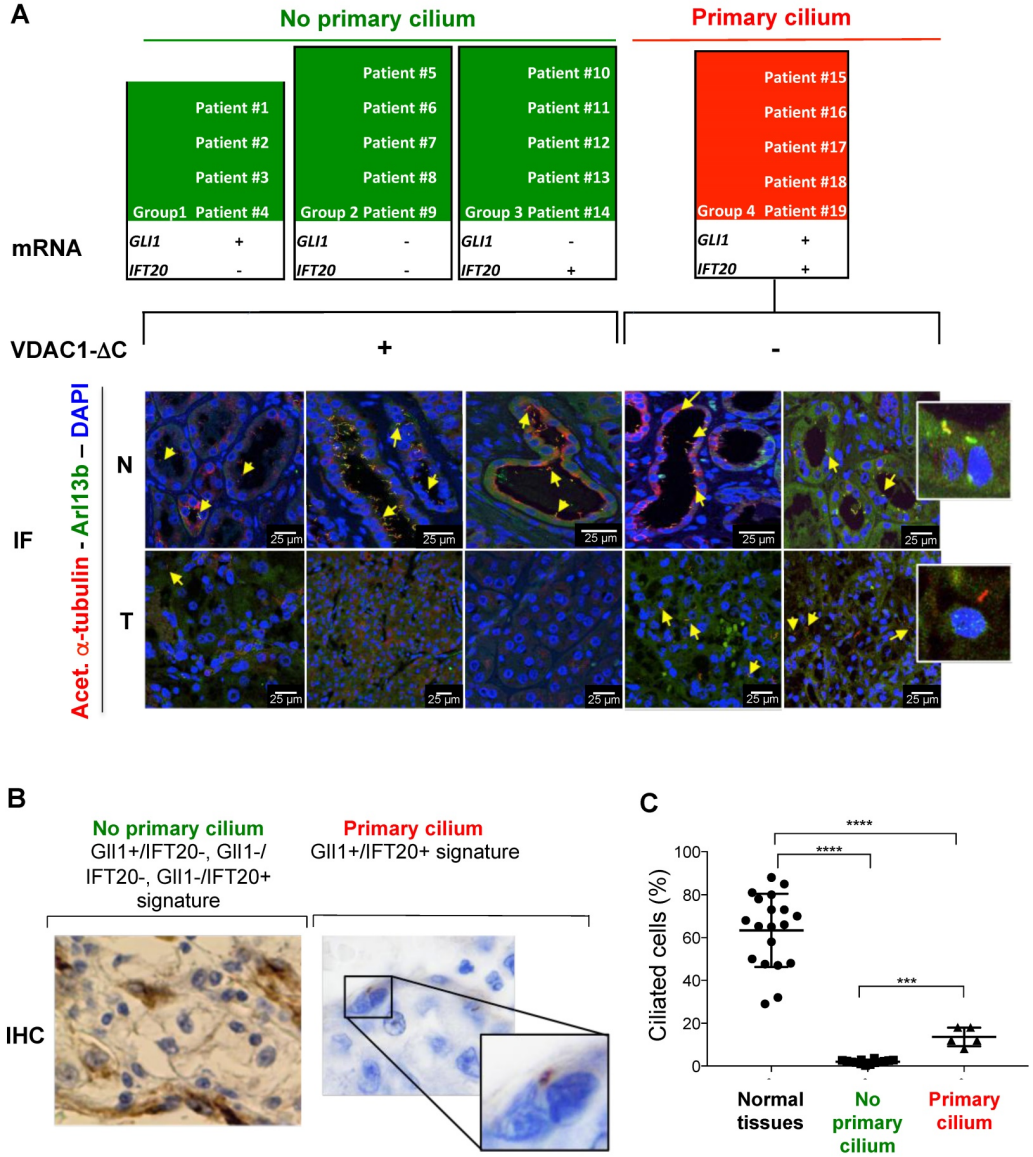
** Identification and validation of the 2-gene signature predictive of the presence of VDAC1-**Δ**C and the primary cilium of ccRCC patients from 19 ccRCC patients. A**, The mRNA (2-gene signature) and immunofluorescence (Acetyl. a-Tubulin, Arl13b and DAPI) of tumors samples of 12 patients were studied to evaluate the prediction model of the absence or presence of the primary cilium. Normal tissues (N) and tumoral tissues (T). **B**, Representative image of immunohistochemistry (IHC) analysis of the 19 patients studied to evaluate a prediction model of the absence or presence of the primary cilium. **C**, Percentage of ciliated cells in normal and tumor tissues from patients. A *** p<0.0005 show significant differences.

To further confirm the value of the *GLI1* and *IFT20* signature obtained *in vitro*, this marker combination was tested on the 19 tissue samples and slides previously described (Figure 1E and Table 1). Four groups were formed on the basis of the expression patterns of these two genes: group 1 with four patients (*GLI1*+/*IFT20*-), group 2 with five patients (*GLI1*-/*IFT20*-), group 3 with five patients (*GLI1*-/*IFT20*+) and group 4 with five patient (*GLI1*+/*IFT20*+; Figure 4A). On the basis of *GLI1* and *IFT20* expression at the mRNA level, but also at the protein level for IFT20 only (Figure S5), and our previous results in ccRCC cell lines, 14 out of 19 (73.7%) from the previous group A, were found to have a “no primary cilium” signature, whereas 5 out of 19 (26.3%) from the previous group B, were classified with a “primary cilium” signature. As expected, we confirmed that the *GLI1*+/*IFT20*-, *GLI1*-/*IFT20*- and *GLI1*-/*IFT20*+ signatures were linked to the presence of VDAC1-ΔC and the absence of the primary cilium, while the *GLI1*+/*IFT20*+ signature was linked to the absence of VDAC1-ΔC and the increased presence of the primary cilium (8-18%; Figure 4A-C).

These results clearly demonstrate that this 2-gene expression signature provides a new form of classification, according to the presence or absence of the primary cilium and depending on VDAC1-ΔC. Moreover, our results unexpectedly uncovered one group of patients in which cancer cells expressed the primary cilium in a ciliopathic disease.

### The tumors in the primary cilium re-expression groups are more aggressive than tumors with ciliopathy

**Figure 5:**
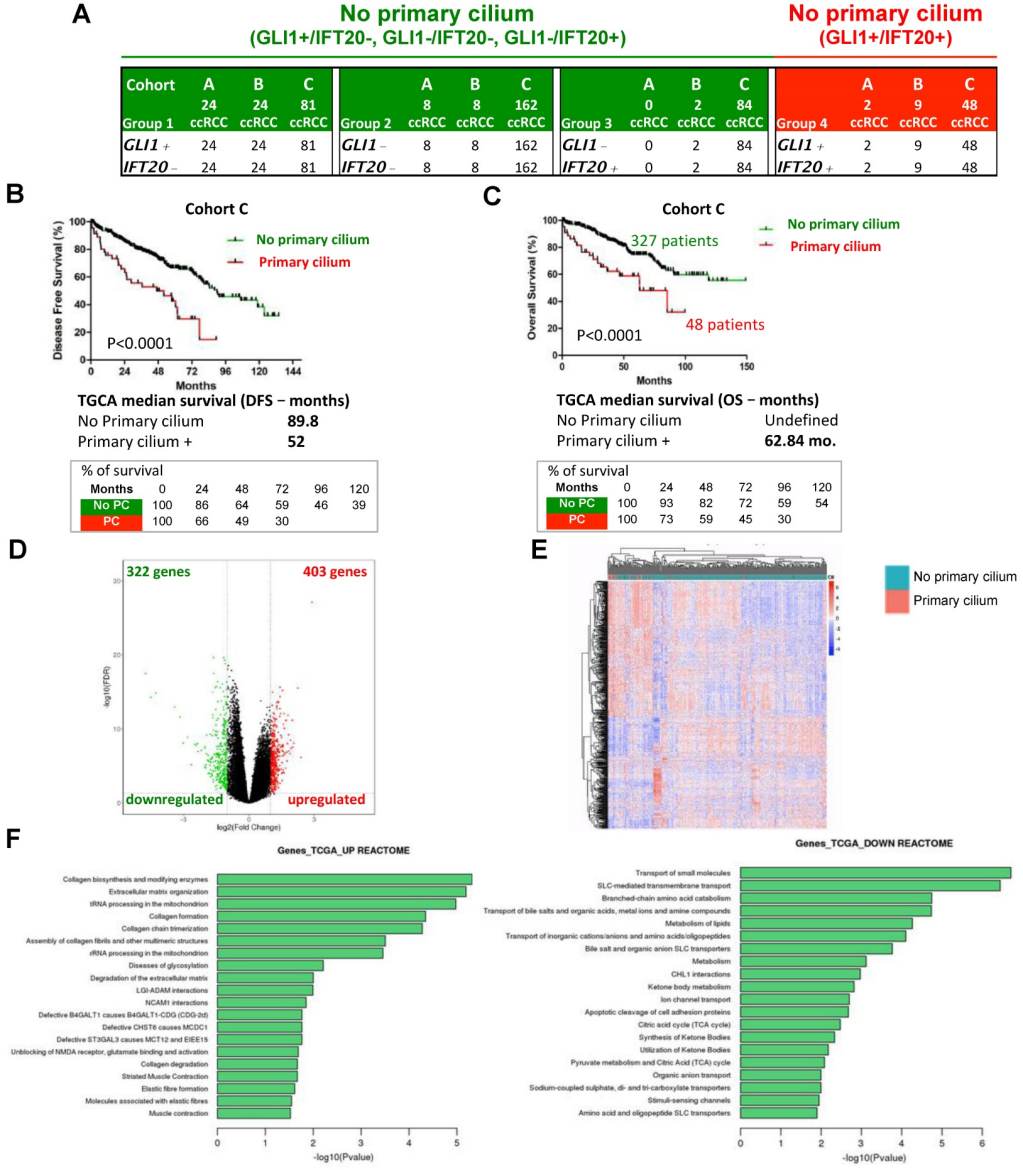
** Identification and validation of the 2-gene signature predictive of the presence of primary cilia and of the aggressiveness of tumors of ccRCC patients from Cohort B and from TCGA (Cohort C). A**, Amount of intra-tumor *GLI1*- and *IFT20*- from the Cohort B and Cohort C. **B**, Disease free survival for the primary cilium signature was calculated from patients of the cohort C using the *GLI1*/*IFT20* signature (less or greater than the third quartile). **C**, Overall survival for the primary cilium signature was calculated from patients of the TCGA cohort C using the *GLI1*/*IFT20* signature (less or grater than the third quartile). **D**, Volcano plot showing the distribution of differentially expressed transcripts. **E**, Heatmap comparing the normalized log2 expression (z score) of the differentially expressed genes between the 48 patients with primary cilium expression and the 327 patients with no primary cilium signature to obtain differentially expressed genes. A Wilcoxon test was performed to obtain a p-value showing the differential significance between the two groups. **F**, Graph of the top 20 enriched Reactome pathways from up-regulated and down-regulated genes.

To investigate the possibility of predicting the prognosis of ccRCC patients based on the presence of the primary cilium, we tested if this two gene signature could be used to classify patients from three cohorts. First, a cohort of patients from PREDIR (Cohort A) was used with 12 patients with hereditary pVHL mutations and 22 sporadic patients presenting tumors with wild-type pVHL and mutated pVHL (Figure S6, Table S2). Tissue samples from 43 patients with non-metastatic ccRCC who had undergone surgery (Urology Department of the Rennes University Hospital; Cohort B; Table S3) 40 and 375 non-metastatic ccRCC tumor samples produced by The Cancer Genome Atlas (TCGA; Cohort C; www.cbioportal.org, TCGA Provisional; RNA-Seq V2) 33 were analyzed. The same four gene signature groups were obtained (Figure 5A). In Cohort A, 100% of the pVHL patients had a signature with no primary cilium (GLI1-/IFT20- and GLI1+/IFT20-), whereas two patients with a primary cilium signature (GLI1+/IFT20+) were found in sporadic patients. No patient presented the group 3 signature in this cohort. For group 4, two (Cohort A) + nine (Cohort B) + 48 (Cohort C) patients were identified. This group represented a low percentage of patients with the primary cilium signature, 5.9%, 20.9% and 12.8% respectively. In Cohort C, *GLI1*+/*IFT20*+ mRNA expression (primary cilium) correlated with shorter DFS (median survival of TCGA, 52 months *versus* 89.8 months (p<0.0001) compared to the *GLI1*+/*IFT20*-, *GLI1*-/*IFT20*- or *GLI1*-/*IFT20*+ signature (no primary cilium; Figure 5B).

Moreover, *GLI1*+/*IFT20*+ mRNA expression also correlated with shorter OS (median survival of cohort B, 101 months versus superior to 150 months (p=0.09) and median survival of TCGA, 62.84 months *versus* greater than 150 months (p<0.0001)) compared to the no primary cilium signature (Figure S7A and Figure 5C), strongly demonstrating that tumors expressing more primary cilia and without VDAC1-ΔC were more aggressive in a ciliopathy model; thus confirming our *in vitro* findings. We found high median survival in both groups 1 and 2 (> 150 months), whereas group 3 presented a lower OS (118.8 months; Figure S7B). We also established a correlation between the primary cilium and VDAC1 (Figure S7C). Patients with no primary cilium expressed a higher level of VDAC1 mRNA, whereas patients with primary cilia expressed a low level of VDAC1 mRNA, similar to the groups A and B that we characterized in Figure 1E. We observed a similar expression level for LGMN mRNA (Figure S7D). Using the tumor proliferation marker KI67 (MKI67) to assess tumor growth, we observed no correlation (p=0.3573) between the absence or the presence of the primary cilium and proliferation, demonstrating that the presence of the primary cilium did not impact the proliferation status of the tumor (Figure S7E). As expected, tumors with stabilization of HIF-2α only presented a tendency to be more aggressive (OS median survival: 72.38 months *versus* undefined, p=0.1035) compared to those expressing both HIF-1α/2α Supplementary (Figure S7F). A volcano plot analysis to further explore the difference between patients with a primary cilium signature and patients with “no primary cilium” signature showed the expression of 403 genes to be UP (1.8%) and 322 DOWN (1.5%) when the mRNAs were significantly differentially expressed (Figure 5D). The results of hierarchical cluster analyses showed distinguishable mRNA expression profiles between the “primary cilium” patients and the “no primary cilium” patients (Figure 5E). Pathway analysis showed that the positively expressed mRNAs in these ccRCC patients were involved in collagen biosynthesis and its modifying enzymes, ECM organization, collagen formation/degradation, degradation of the ECM, whereas the negatively expressed mRNAs were involved in transport of small molecules, solute-carrier-mediated transmembrane transport, metabolism of lipids, and the TCA cycle. The UP mRNA signature pathways strongly suggested involvement of EMT (epithelial-mesenchymal transition; Figure 5F), which was confirmed by analyzing the expression patterns of individual genes such as *SNAIL*, *SLUG*, *TWIST1*, *TWIST2*, *TBX2* and *FN1* (Figure S8). However, the DOWN mRNAs indicated complete metabolic reprogramming (Figure 5F). An in depth analysis of the TCGA database revealed 310 patients with pVHL- ccRCC and 65 patients with pVHL+ ccRCC defined *via* deletions, mutations and promoter methylation in pVHL (Figure S9). Among the pVHL- tumors, 250 ccRCC tumors in the TCGA expressed both HIF-1 and -2 but 60 expressed only HIF-2. Using the primary cilium signature, we characterized two sub-groups, no primary cilium (PC-) and primary cilium (PC+) in each category. The PC+ group contained a low number of patients (10.4%, 21.7% and 13.8%) compared to the PC- group and showed lower median survival. We also found that the majority of PC+ patients were mostly at advanced tumor stages (stage 3/4-66%) rather than stage 1/2 (34%), whereas we observed the opposite with PC- patients, 25.2% and 74.8% respectively (Table S4). Group 4 of PC+ patients presented a more aggressive pattern with increases in pathways for extracellular matrix modifications coupled with decreased OXPHOS and lipid metabolism but maintenance of glycolysis, which would favor EMT.

Finally, *in silico* transcriptomic data showed that the primary cilium signature correlates with tumor stage and, to a lesser extent, with the Furhman grade (Table 1A). The “primary cilium” signature, Furhman grade, tumor stage and age have an impact on DFS and OS (univariated analysis, Table 1B). The “primary cilium” signature represented a marker for DFS (Table 1C) and OS (Table 1D) independent of the tumor stage and age in a multivariate analysis. As an example, hazard ratios show that PC+ patients will be twice as metastatic as PC- patients (Hazard ratio = 2.448, DSF; Table 1C) and will die faster (Hazard ratio = 2.13, OS; Table 1D).

These results highlight that tumors from ccRCC patients expressing primary cilia with a *GLI1*+/*IFT20*+ signature but not VDAC1-ΔC are significantly more aggressive and are characterized by a poor prognosis. In this context, the increased presence of primary cilia in tumors is clearly a cancer promoter and the maintenance of glycolysis seems to be crucial to support this aggressiveness.

### The tumors of primary cilium re-expression groups should respond to anti-glycolysis treatments and have a higher score indicative of better response to immunotherapy

**Figure 6:**
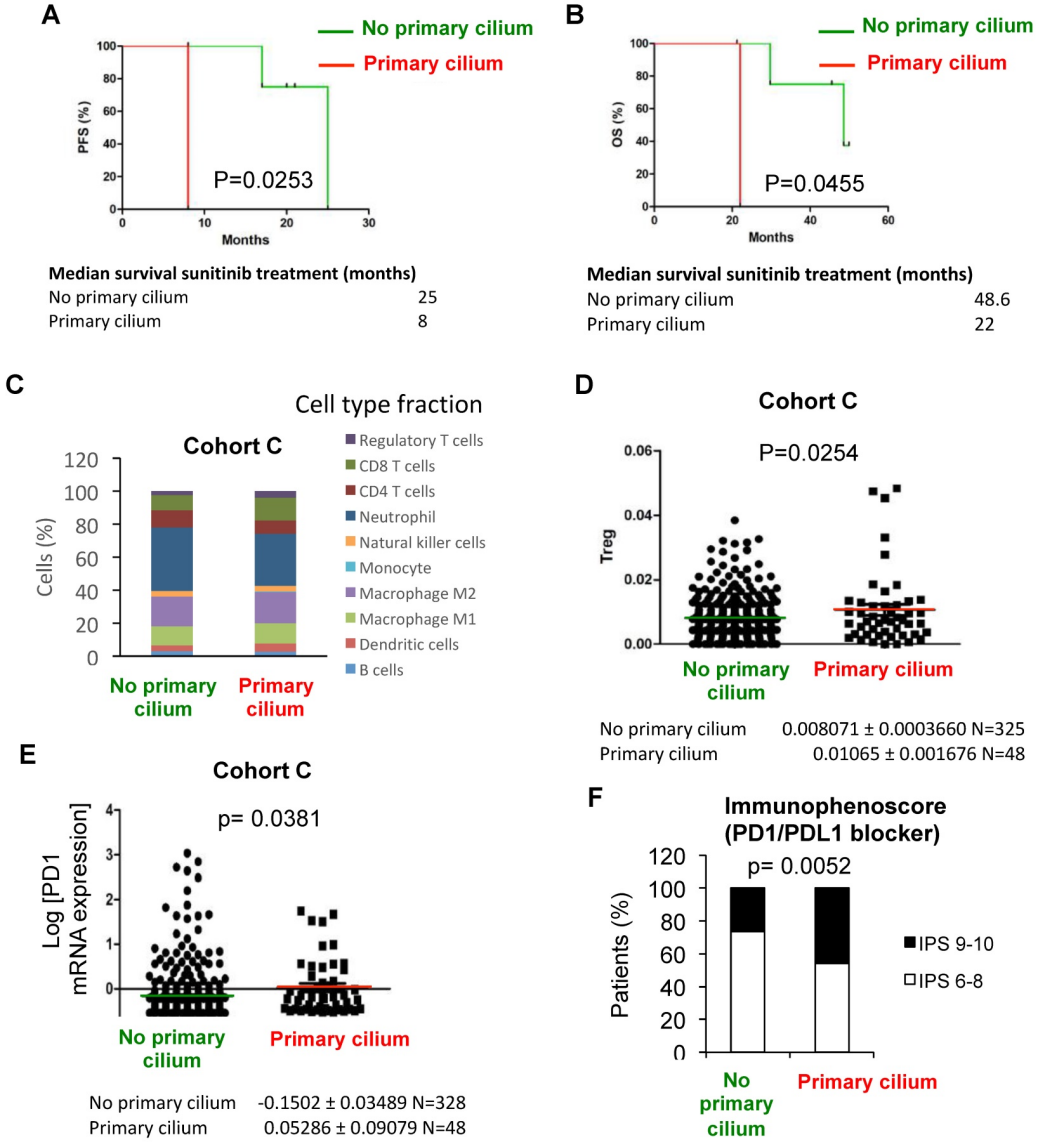
** Patients with primary cilium signature present a higher immunogenicity compared to no primary cilium signature and a better response to immunotherapy. A and B**, Progression free-survival (PFS) (**A**) and Overall survival (OS) (**B**) for the primary cilium signature was calculated from patients of the TCGA cohort C treated with sunitinib using the *GLI1*/*IFT20* signature. PFS and OS were calculated from patient subgroups with *GLI1*/*IFT20* mRNA levels that were less or greater than the third quartile. Statistical significance (p-value) is indicated. The median survival is also indicated. **C**, *In silico* analysis of immune cell type fraction (%) according to the primary signature status (no primary cilium and primary cilium). **D**, *In silico* analysis of regulatory T lymphocytes (T reg) fraction (%) according to the primary signature status (no primary cilium and primary cilium). **E**, Tumors from patients with no primary cilium signature and tumors from patients with primary cilium were compared. The level of PD1 mRNA was determined by RNAseq for the TCGA cohort. Statistical significance (p value) is indicated. **F**, Distribution of ccRCC patients from the TCGA database depending on the primary cilium signature and immunophenoscore. p-value between no primary cilium and primary cilium is indicated.

Sunitinib (sunitinib malate, Sutent^®^, SU11248, Pfizer Inc.), a vascular endothelial growth factor receptor inhibitor, is widely used for patients with metastatic RCC, mostly in first-line treatment. Despite sunitinib's clinical efficacy, patients eventually develop drug resistance and disease progression 41-45. We thus checked if patients with tumors presenting the primary cilium, a *GLI1*+/*IFT20*+ signature and no VDAC1-ΔC are resistant or sensitive to sunitinib. Analysis of these patients from the clinical trial SUVEGIL (Clinical trials.gov Identifier: NCT00943839), who had been treated with sunitinib, revealed that they had a lower survival rate than the groups with no primary cilium (Figure 6A and B, Table S5). To reinforce these results, *in vitro* experiments were conducted using RCC4 and RCC4+pVHL cells in the absence or presence of sunitinib. We found that cells with VDAC1-ΔC were significantly more sensitive to treatment than RCC4+pVHL cells (Figure S10A). RCC4-, RCC4 siVDAC1- and RCC4 siLGMN- cells were also treated with 5 µM of sunitinib (Figure S10B). Although sensitive to sunitinib, we found that RCC4 cells with VDAC1-ΔC had a lower percentage of ciliated cells and were characterized by a “no primary cilium” signature, and were slightly more sensitive to treatment than cells with less VDAC1 or no VDAC1-ΔC with a higher percentage of cilia and with a “primary cilium” signature. This was similar to the observations of patients from group 4 with more primary cilia.

As group 4 of “primary cilium” patients present a decrease in OXPHOS and lipid metabolism but maintenance of glycolysis, we used 3 Bromopyruvate (3BP), a halogenated analog of pyruvic acid that enters cells in the same way as lactate molecules, *via* monocarboxylic acid transporters, to block glycolysis in *in vitro* experiments. As expected, RCC4+pVHL cells and RCC4 siVDAC1 or RCC4 siLGMN with no VDAC1-ΔC, all characterized by an increase in primary cilia expression, were highly sensitive to 25 µM of 3BP compared to RCC4 or RCC4 siCtl (Figure S10C and D). Moreover, the aggressiveness of RCC4 siVDAC1 or siLGMN treated with 3BP was decreased compared to the control (Figure S10E and F), suggesting a potential therapeutic use of glycolysis inhibitors.

Finally, as immunotherapy has become increasingly common for the treatment of clear cell RCC, we investigated the immune profile for each group. Slight differences were observed in the relative fraction of major immune cell types in “primary cilium” patients compared to “no primary cilium” patients of the TCGA cohort (Figure 6C) and a significantly higher proportion of T regulatory lymphocytes (Treg) was observed in the “primary cilium” patient group (Figure 6D). To reinforce these results, PD1 mRNA expression was significantly increased in the “primary cilium” patient group (Figure 6E). Furthermore, the immunophenoscore (Figure 6F), used as a predictor of response to anti-programmed cell death protein 1 (anti-PD1) treatment, was favorable for the “primary cilium” patient group. In the analysis of groups A and B from Figure 1G, we also observed a significant increase of PD-L1 mRNA expression in the “primary cilium” patient group B (Figure S11A) associated with increased frequency of cytoplasmic PDL1 punctae within vesicle-like structures in patient tumor sections (Figure S11B). These results clearly showed the specific immunotherapeutic potential for the “primary cilium” patient group.

Considered together, these results suggest that sunitinib is not the best treatment for patients re-expressing primary cilium. Our results indicate two therapeutic approaches, glycolysis inhibitors (3BP) and/or anti-PD1, that could be used in the first or second line only for the patients that present the strong “primary cilium” signature and for whom sunitinib is unfortunately less effective.

## Discussion

Our data describe i) a new mechanism for the control of ciliogenesis that is driven by VDAC1-ΔC, the form of VDAC1 that is produced in hypoxia and ii) a new group of ccRCC patients in which the primary cilium is re-expressed, giving rise to greater tumor aggressiveness.

The role of VDAC in metabolic homeostasis and cell death has been studied extensively 19, 20, 46-54. However, new functions for VDAC1 as a ciliogenesis controller have been discovered, with Majumder *et al.* recently showing that centrosomal VDAC3 is associated with the centrosome *via* Mps1, a protein kinase that plays a role in centriole assembly 24. The Mps1-VDAC3 complex, and also centrosomal VDAC1, were involved in the negative regulation of ciliogenesis. However, these results are not directly comparable because Majumder *et al.* used a retinal cell model rather than cancer cell models and we studied mitochondrial VDAC1 instead of centrosomal VDAC1. Our preliminary results have shown that VDAC1 is in close proximity to the centrosome (data not shown) suggesting that VDAC1 could directly participate in ciliogenesis, a mechanism that we are exploring further. We focused on the role of VDAC1-ΔC formation and the involvement of HIF-1. We have previously shown that VDAC1-ΔC formation is mitochondrial and is dependent on nuclear HIF-1α in a lung cancer model 22. However, it has been demonstrated that VDAC1-ΔC formation can also be triggered by the physical association of HIF-1α with the mitochondrial outer membrane and a mortalin/VDAC1/HK2 complex under conditions that inhibit ERK activity and HIF-1α phosphorylation 55. Thus, we checked the subcellular localization of HIFs in RCC4 and the ERK activation status in patients. Our data clearly showed that HIFs are present in the nucleus of the RCC4 cells, as previously observed in LS174 cells, whereas P-ERK1/2 was observed in both Group A and Group B (data not shown) strongly suggesting that this mechanism is cell-type specific and does not occur in our ccRCC model.

Since 2012, we have been investigating the role of VDAC1-ΔC in hypoxia 22, 23, 27 and under iron deprivation conditions 56. Our study describes, for the first time, a VDAC1-ΔC-dependent mechanism in which kidney cancer cells can maintain glycolysis in the presence of the EMT signature, which promotes survival of cells surrounded by an unfavorable microenvironment. Our study definitively shows that the hypoxic 2-gene expression signature, which we characterized, is closely related to the formation of VDAC1-ΔC and the absence or decreased prevalence of the primary cilium. The global OS allowed us to classify patients, by differentiating levels of tumor aggressiveness. However, we also described a new group of patients (group 4: *GLI1*+/*IFT20*+) expressing/re-expressing the primary cilium in a ciliopathy context. We demonstrated that patients belonging to this group had much more aggressive tumors than patients with few or no primary cilia. The tumors in this group could be directly derived from healthy tissue but the expression of the EMT genes made the tumors of these patients more aggressive. In the first cohort we studied (Cohort A-PREDIR), the *GLI1*+/*IFT20*+ signature was observed only in sporadic patients, suggesting that such expression/re-expression was impossible in patients with VHL mutations at the germinal level. However, the cohort of VHL patients was too small to draw conclusions.

Interestingly, we also found that each group (pVHL-/HIF-1+/HIF-2+, pVHL-/HIF-2+ and pVHL+) from the TCGA database presented 10.4, 21.7 and 13.8% of patients, respectively, with a *GLI1*+/*IFT20*+ signature. The median survival of these patients was lower than for patients with a “no primary cilium” signature. Moreover, patients with this *GLI1*+/*IFT20*+ signature presented a significant correlation between aggressiveness and lower VDAC1 expression. Similarly, in our study, siRNA to VDAC1 and LGMN in RCC4 cells shifted the signature in *GLI1*+/*IFT20*+ and increased the primary cilium expression. These cells were characterized by higher invasion than siRNA to control cells suggesting a more aggressive phenotype. The same invasive behavior was found in RCC4+pVHL cells. The tumor aggressiveness in these patients could be the result of the combination of a switch to EMT process activation, re-expression or maintenance of the primary cilium, together with a decrease in VDAC1 and LGMN expression and thus the disappearance of the cleaved form of VDAC1.

In patients who express/re-express the primary cilium, it is unlikely that the primary cilium is the only force associated with aggressiveness. Indeed, we characterized a signature related to EMT that can explain the aggressive phenotype of the tumors of these patients. Moreover, metabolic remodeling was impacted, and although cancer metabolism is a hallmark of cancer, it has been shown that aberrant metabolism supports EMT 57, 58. In the signature of the present study, lipid metabolism and the TCA signature were down-regulated in line with observations made by Hakimi *et al.* for ccRCC patients 59. However, the expression of glycolytic enzymes was not modified, strongly suggesting that this metabolic pathway was favored in the cancer cells of group 4 patients. It is therefore possible to envisage specific treatment for these groups: Temsirolimus 60 or Everolimus, specific inhibitors of mTOR 61 that block proliferation, in combination with small molecule inhibitors that prevent EMT such as EW-7197 or IN-1130, through a block in TGFβ 1 and 2, have already been used in metastatic breast and lung cancer 62. By maintaining only one metabolic pathway, cancer cells with a *GLI1*+/*IFT20*+ signature offer a metabolic vulnerability that we would be wise to exploit. We have shown that inhibitors of glycolysis such as 3-bromopyruvate, used as a proof-of-concept, or inhibitors of lactate production (dichloroacetate, FX11, AZD-3965) 58 are of interest.

Finally, an immune-checkpoint inhibitor such as atezolizumab or nivolumab (an anti-PD-L1 or PD1 inhibitor) alone or in combination with glycolysis inhibitors could be evaluated on “primary cilium” patients who exhibit such reduced overall survival. Indeed, the presence of PD-L1 in group B strongly suggests an important role in promoting tumor progression. We have proposed several hypotheses to explain such expression patterns. Firstly, Noman and Chouaib have revealed the binding of HIF-1α to the PD-L1 promoter 63, although we showed that absence of the primary cilium is driven by the pVHL/HIF/hypoxia axis, we showed that the resurgence of primary cilia in patients from group 4 was independent of HIF-1, HIF-2 and pVHL. Secondly, it has been reported that PDL1 works predominantly in lactate-enriched tumor microenvironments 64. As tumors of patients from group 4 maintain glycolysis, and thus lactate production, this suitable microenvironment may protect cancer cells and thus could participate in the activation of PD-L1. Thirdly, epigenetic regulation has been revealed to be involved in PD-L1 expression in cancer cells 65. MiRs, P53 and STAT3 were reported to epigenetically regulate PD-L1 expression. As ccRCC patients are mainly p53 wild type, we focused on STAT3 and observed a significant (p<0.001) increase in STAT3 expression in patients from group 4 (data not shown). Understanding what regulates PDL1 in patients expressing primary cilia will be thus an important avenue of research going forward.

This novel classification of *GLI1*+/*IFT20*+ ccRCC patients should have an impact on clinical practice, not only in characterizing new subgroups of ccRCC patients, but also in offering new combinations of treatments that are much more effective and more specific for a specific group of ccRCC patients.

## Supplementary Material

Supplementary figures and tables.

## Figures and Tables

**Table 1:**
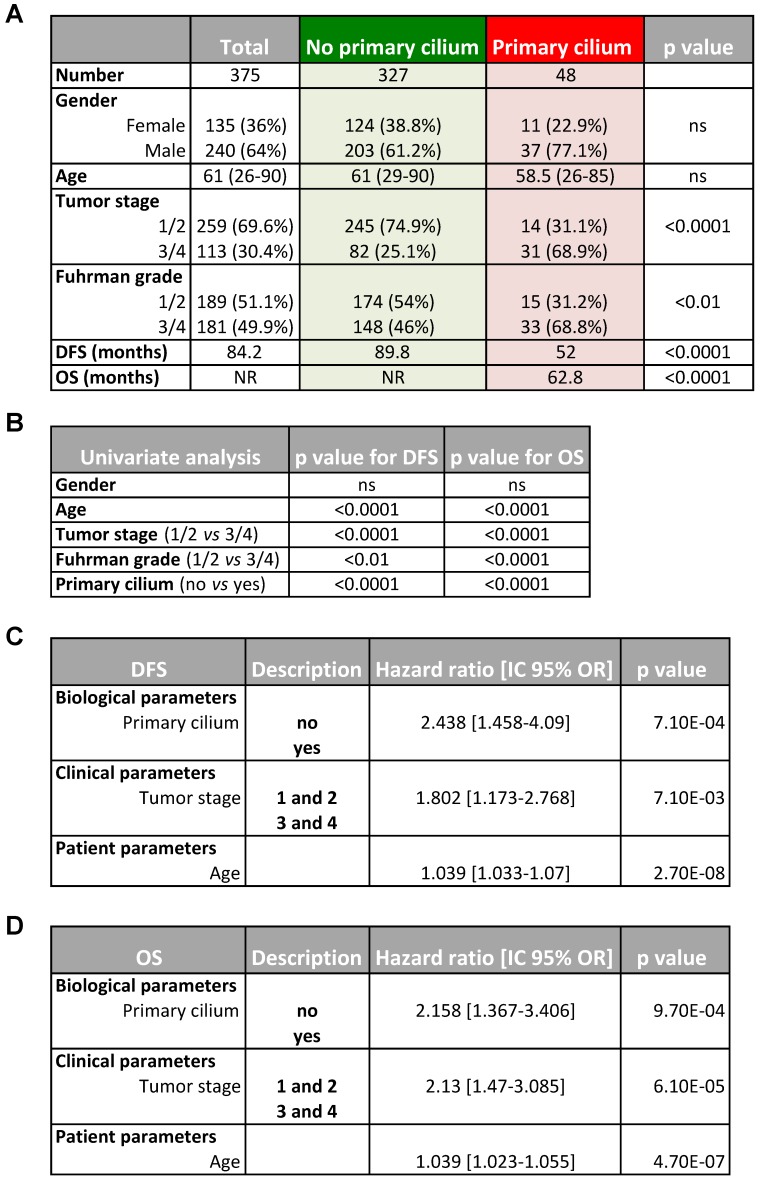
Patient characteristics included in the study and multivariate analysis.

**A**, Patient characteristics and univariate analysis with the Fisher or Ki2 test. Statistical significance (*p* values) are indicated. **B**, Univariate analysis of primary cilium, tumor stage, Furhman grade, age and PFS or OS. Statistical significance (*p* values) is indicated. **C and D,** Multivariate analysis of primary cilium, tumor stage, age and PFS (**C**) or OS (**D**). The multivariate analysis was performed using Cox regression adjusted to the tumor stage and age. Statistical significance (*p* values) is indicated.
