# Supplementary material for: Identification of a new aggressive axis driven by ciliogenesis and absence of VDAC1-ΔC in clear cell Renal Cell Carcinoma patients
Source: Theranostics. 2020 Feb 3;10(6):2696–713. doi: 10.7150/thno.41001 (PMC7052902; doi:10.7150/thno.41001)
Supplement: Supplementary file 1 — Supplementary figures and tables. [file thnov10p2696s1.pdf]

A

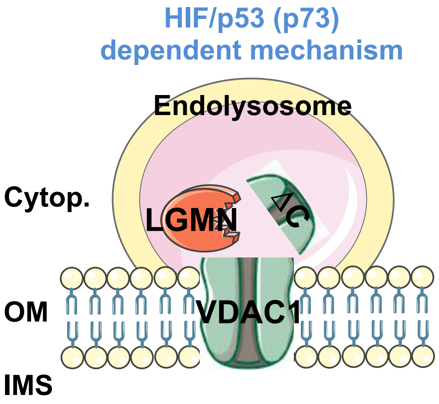

B

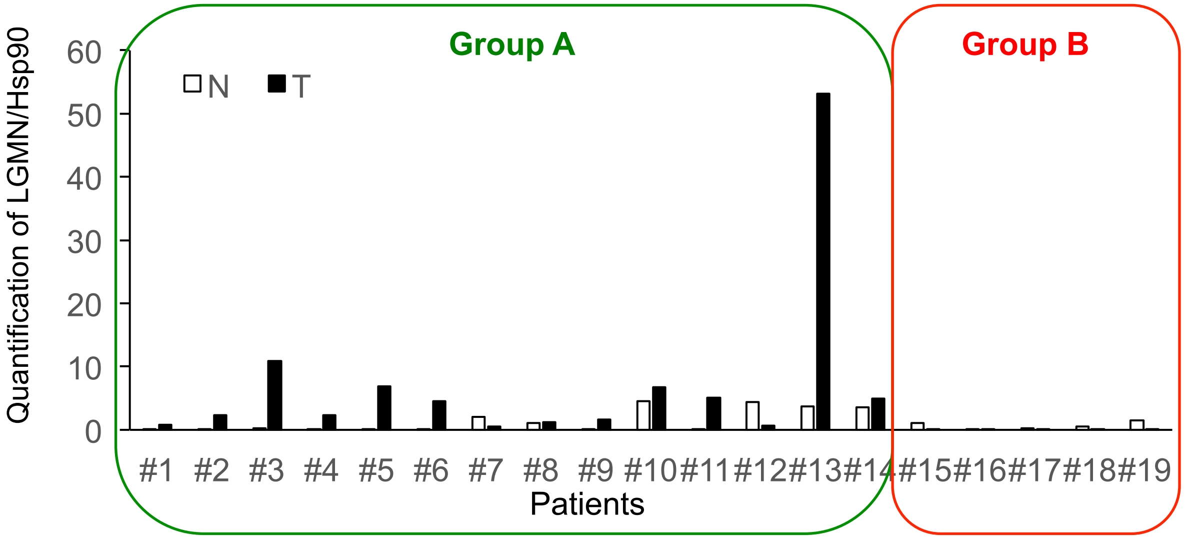

C

Patient's number	#1	#2	#3	#4	#5	#6	#7	#8	#9	#10	#11	#12	#13	#14	#15	#16	#17	#18	#19
HIF1 statut (T/N CAIX expression)	+	+	/	+	−	+	+	+	+	−	−	−	−	+	+	+	−	+	+
HIF2 statut (T/N Oct4 expression)	+	+	/	+	+	+	+	+	+	+	+	+	+	+	+	+	+	+	+

Figure S1. Legumain, a direct partner of the cleaved form of VDAC1. A, Schematique representation of VDAC1 cleavage by LGMN. Cytoplasm (Cytop.), mitochondrial outer membrane (OM), mitochondrial intermembrane space (IMS). **B,** Expression of LGMN and Hsp-90 proteins was quantified in normal (N) and tumoral (T) tissues and LGMN/Hsp-90 ratio was obtained in each patient. **C**, Representative table of HIF status depending on the mRNA level of CAIX (HIF-1 signature) and Oct4 (HIF-2 signature) in patients from group A and Group B.

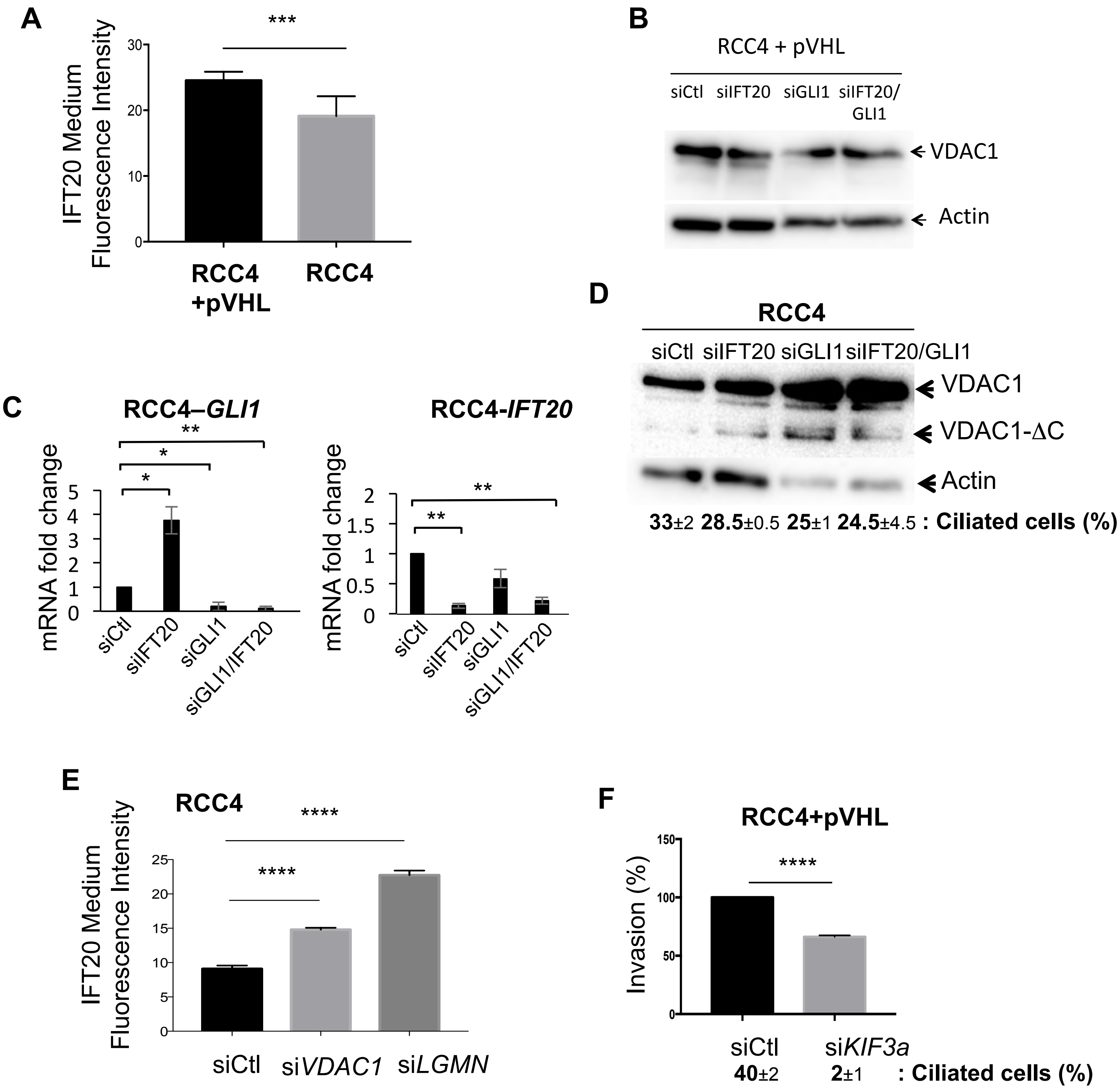

Figure S2. GLI1/IFT20 signature is correlated to primary cilium and VDAC1 in RCC4 cells. A, IFT20 medium fluorescence analysis by FACS in RCC4+pVHL and RCC4 cells. **B**, RCC4+pVHL cells were transfected with control siRNA (siCtl), siIFT20, siGLI1 and siIFT20/GLI1. Cell lysates were analyzed by immunoblotting for VDAC1 and Actin was used as a loading control. **C**, Histograms represent the expression of the mRNA of *IFT20* (left panel) and *GLI1* (right panel) in RCC4 cells in Nx. **D,** RCC4 cells were transfected with control siRNA (siCtl), siIFT20, siGLI1 and siIFT20/GLI1. Cell lysates were analyzed by immunoblotting for VDAC1 and Actin was used as a loading control. Quantitative analysis of the ciliation percentage was assessed by confocal fluorescence microscopy (n=100-300 cells). **E,** IFT20 medium fluorescence analysis by FACS in RCC4 cells transfected with control siRNA (siCtl), siVDAC1 and siLGMN. **F**, Graphic representation of the Boyden chamber cell-based invasion assay using RCC4+pVHL cells transfected with siRNA *KIF3A* compared to Ctl (siCtl). The mean ±SEM is representative of three independent experiments.

A * $p<0.05$, ** $p<0.005$ and **** $p<0.0005$ show significant differences. Experiments have been proceeded without serum.

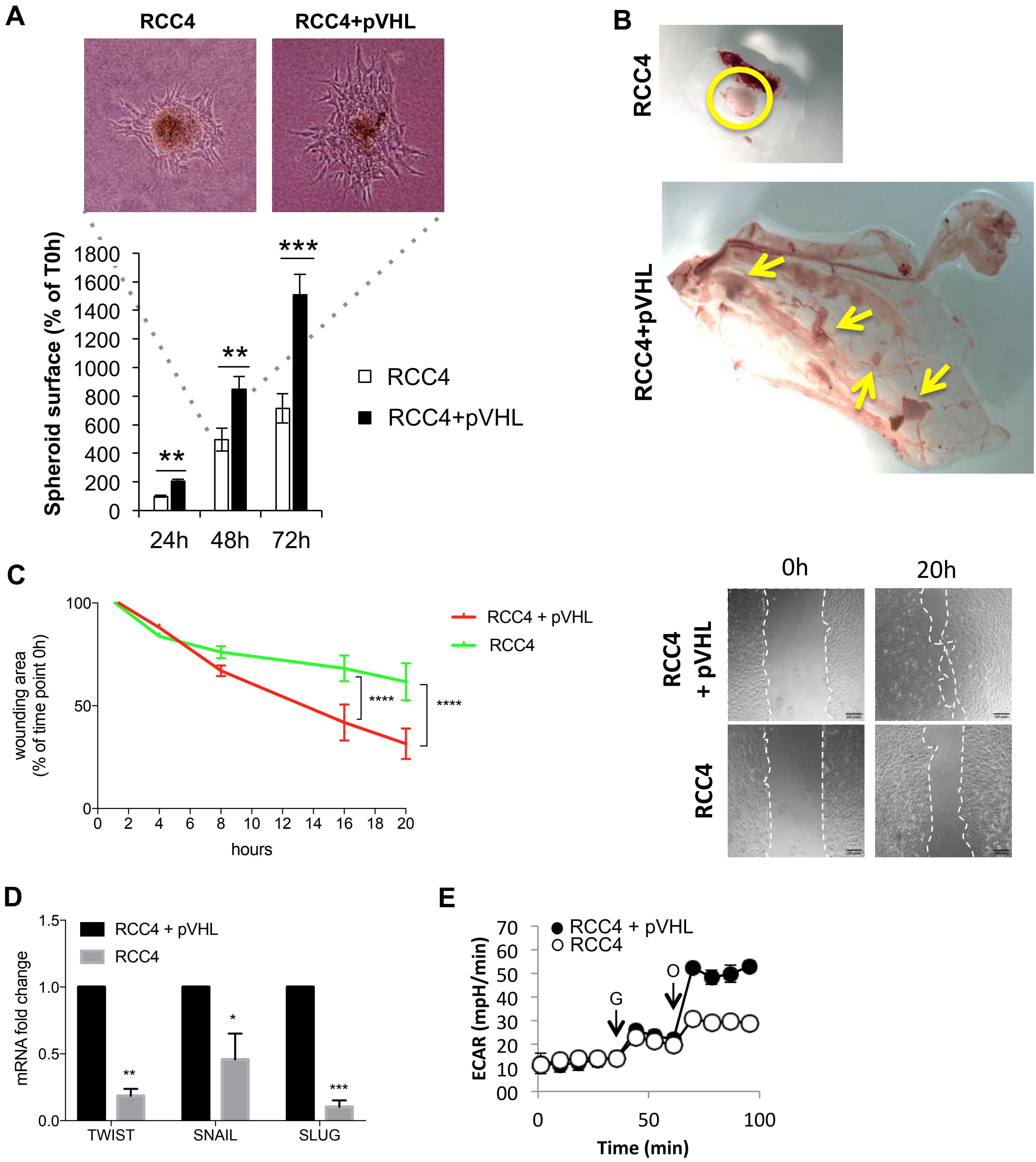

Figure S3. RCC4+pVHL cells are more aggressive than RCC4 cells. A, Tumor spheroid invasion assay was performed with RCC4 and RCC4+pVHL. *Insert*, brightfield of RCC4 and RCC4+pVHL spheroids afeter 48h. Percentage (%) change in RCC4 and RCC4+pVHL spheroids'surfaces within 48h compared to T0h. At least 8 spheroids were analyzed per condition and 3 independent experiments were performed. A ** p<0.005 and **** p<0.0005 show significant differences. **B**, Shell-less chick embryo with RCC4 microtumors only (circles) and RCC4+pVHL micrometastasis (arrows) developed for 7 days after grafting cells withing a drop of matrigel. **C**, Wound-healing assays. **D**, Histograms represent the expression of the mRNA of *TWIST, SNAIL and SLUG* in RCC4 and RCC4+pVHL cells in Nx. **E**, The extracellular acidification rate (ECAR) RCC4+pVHL and RCC4 cells in Nx was evaluated with a Seahorse XF bioenergetic system. Cells were deprived of glucose for 1h (basal), then glucose (10mM), to measure glycolysis and oligomycin (1µM – glycolytic capacity) to measure the full glycolytic capacity, were injected. The mean ± SEM is representative of two independent experiments carried out in quadruplicate.

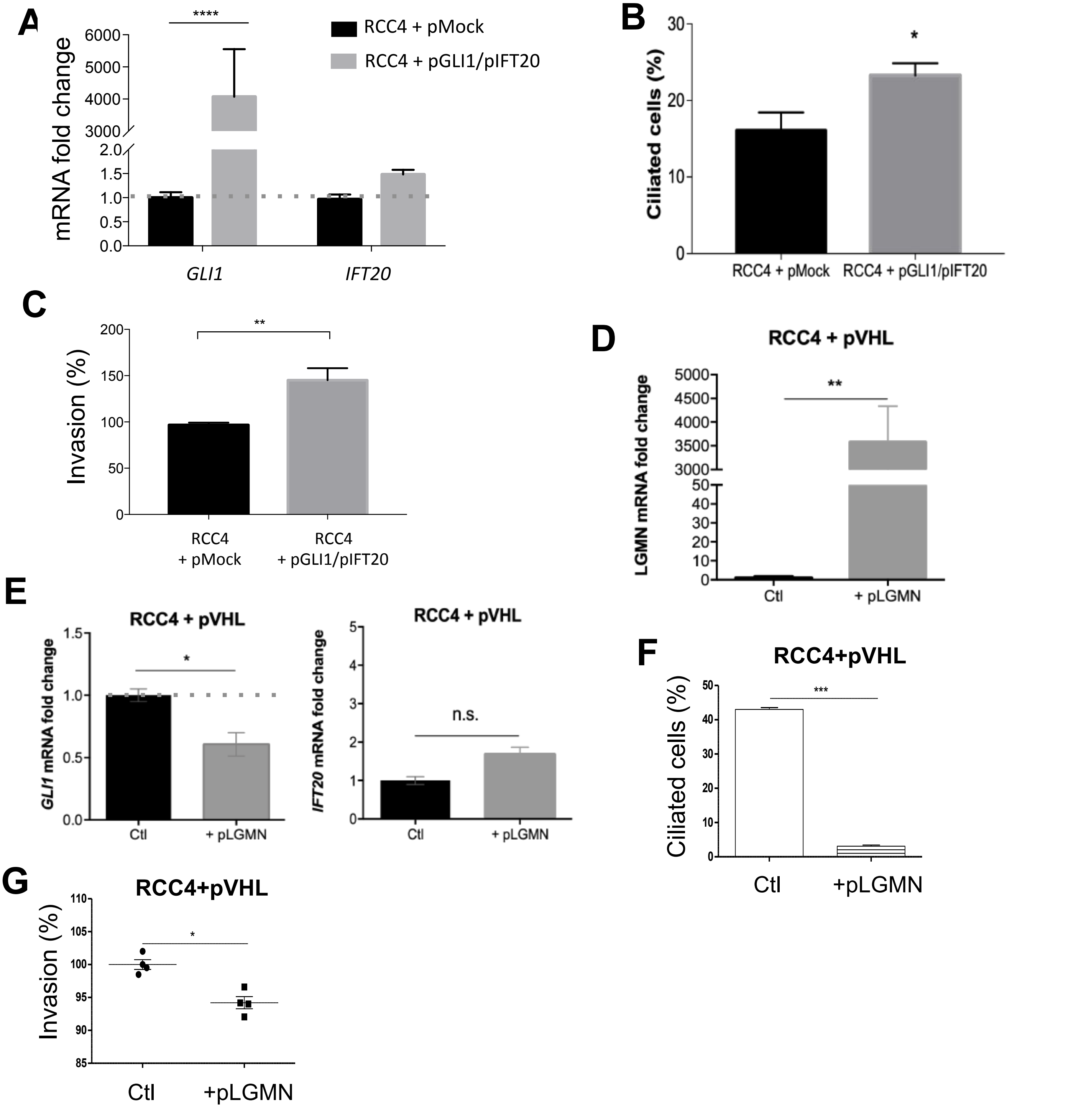

Figure S4. Modulation of GLI1/IFT20 or LGMN is correlated to primary cilium in RCC4 or RCC4+pVHL cells. A, Histograms represent the overexpression of *GLI1* (pGLI1) and *IFT20* (pIFT20) at the mRNA level in RCC4 cells in Nx pMock reprents the empty vector. **B,** RCC4 cells were transfected with control vector (pMock) and pIFT20/GLI1. Quantitative analysis of the ciliation percentage was assessed by confocal fluorescence microscopy (n=100-300 cells). **C**, Graphic representation of the Boyden chamber cell-based invasion assay using RCC4 cells transfected with pGLI1/pIFT20 compared to Ctl (pMock). The mean ±SEM is representative of three independent experiments. **D,** Histograms represent the overexpression of *LGMN* (pLGMN) at the mRNA level in RCC4+pVHL cells in Nx. **E,** RCC4+pVHL cells were transfected with control vector (Ctl) and pLGMN. mRNA expression of *GLI1* (left panel) and *IFT20* (right panel) was quantified in RCC4 cells. **F**, RCC4+pVHL cells were transfected with control vector (Ctl) and pLGMN. Quantitative analysis of the ciliation percentage was assessed by confocal fluorescence microscopy (n=100-300 cells). **G**, Graphic representation of the Boyden chamber cell-based invasion assay using RCC4+pVHL cells transfected with pLGMN compared to Ctl. The mean ±SEM is representative of three independent experiments.

A * p<0.05, ** p<0.005 and **** p<0.0005 show significant differences. Experiments were conducted without serum.

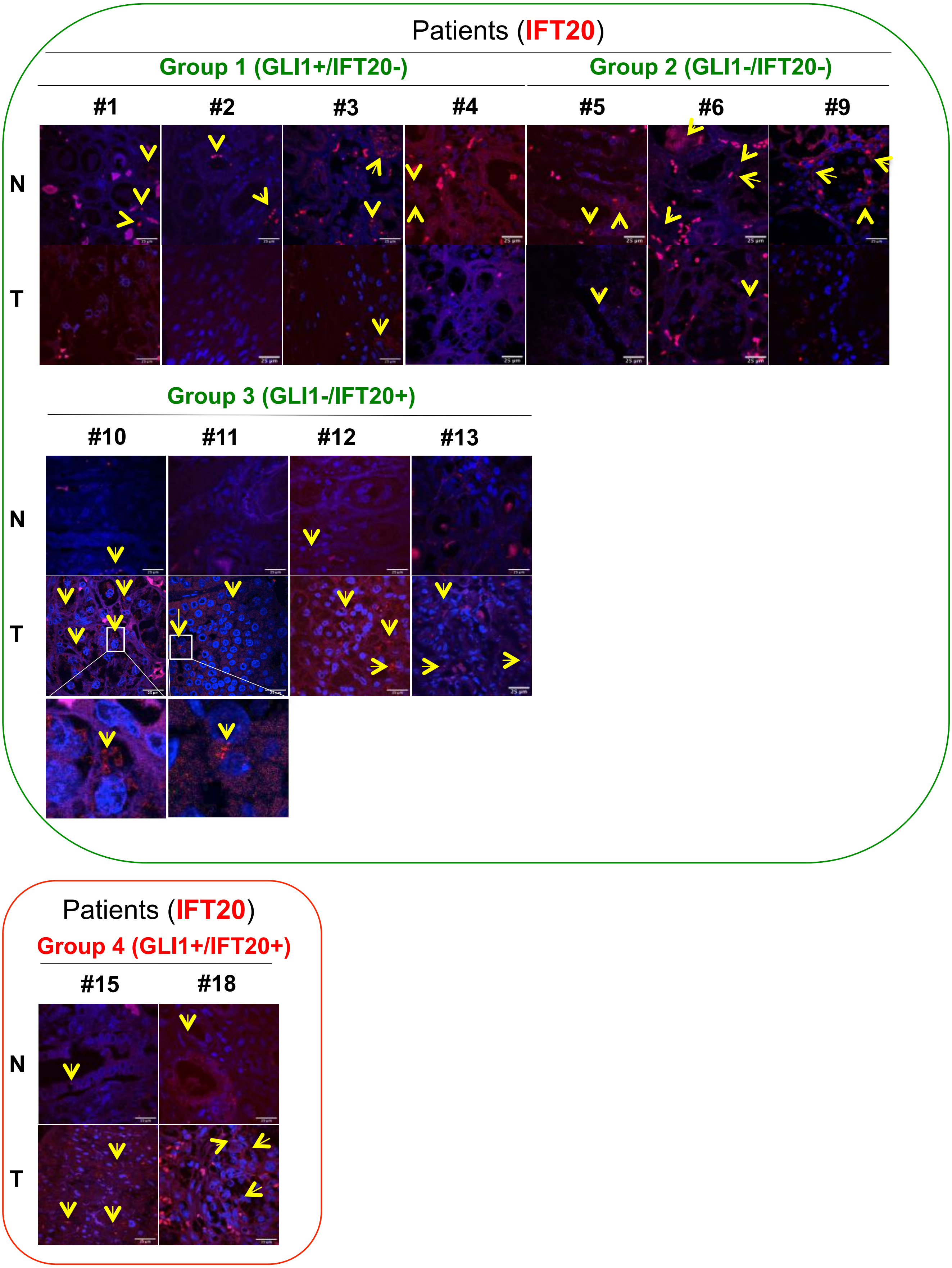

Figure S5. IFT20 protein expression in patients. Representative image of immunohistochemistry (IHC) analysis of IFT20 expression of the 13 patients out of 19 studied to evaluate a prediction model of the absence or presence of the primary cilium.

A

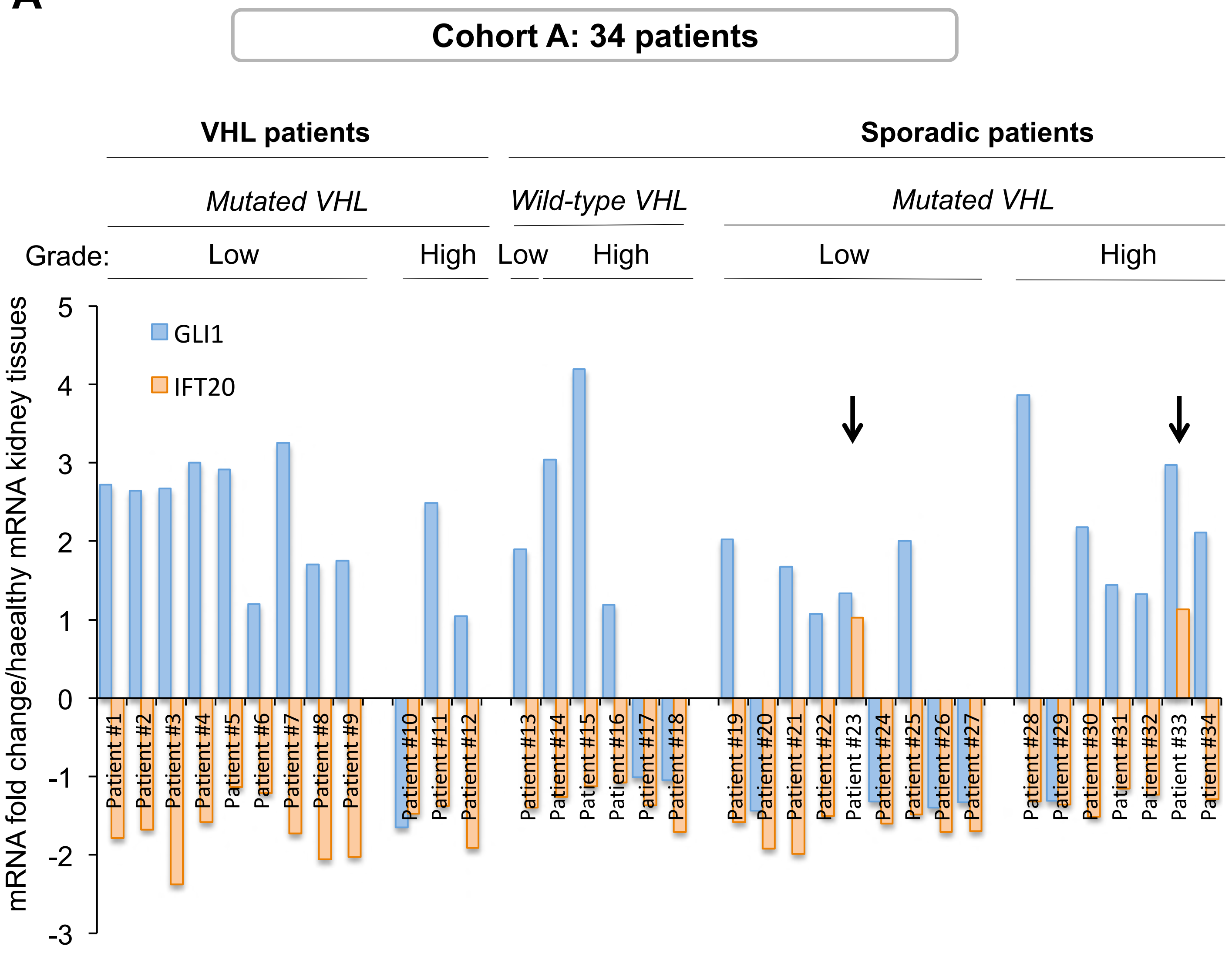

B

Patients #	1	2	3	4	5	6	7	8	9	10	11	12	13	14	15	16	17	18	19	20	21	22	23	24	25	26	27	28	29	30	31	32	33	34
**HIF-1**	+	+	+	+	+	+	+	+	+	+	+	+	+	-	-	+	-	+	+	+	-	-	+	+	-	+	+	-	+	+	+	+	-	+
**HIF-2**	+	+	+	+	+	+	+	+	+	+	+	+	+	+	+	+	+	+	+	+	+	+	+	+	+	+	+	+	+	+	+	+	+	+

Figure S6. Cohort A classification. A, Amount of intra-tumor GLI1- and *IFT20* mRNA in VHL- and sporadic patients from cohort A. These two groups were separated depending on whether they presented the wild-type or mutated form of pVHL and each of these sub-groups were divided depending on a low or high grade of ccRCC. The mRNA fold change of patients is compared to a pool of healthy kidney tissues.**B**, HIF-1 and HIF-2 status of the 34 patients from cohort A was obtained by immunohistochemistry using HIF-1α and HIF-2α antibodies and confirmed with the expression pattern of HIF-1/HIF-2 target genes, *ca9/bnip3* and *pouf5F1/slc2a1*, respectively.

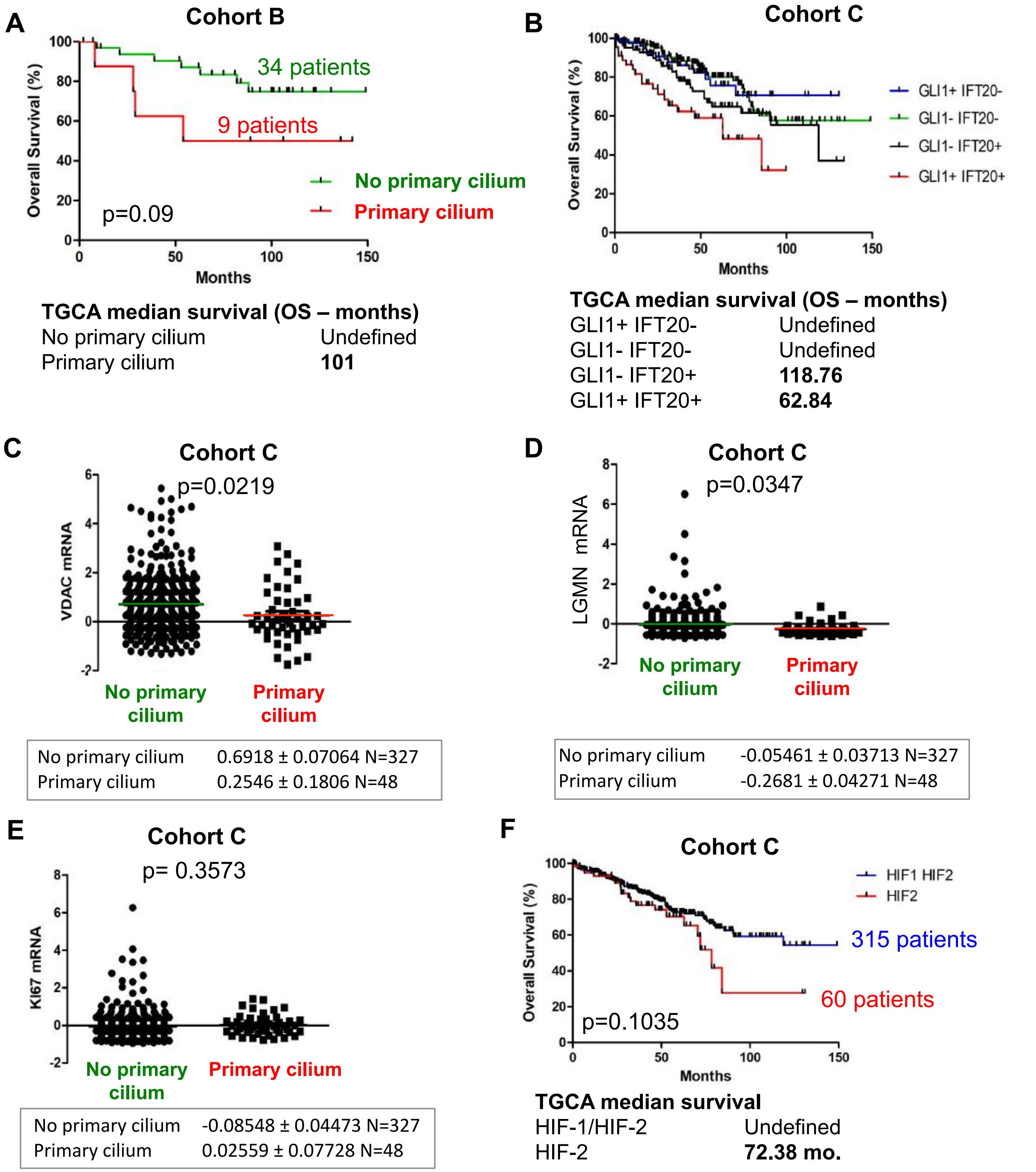

Figure S7. Characterization of the 375 patients from the TCGA ccRCC cohort according to the primary cilium status. A, Overall survival for the primary cilium signature was calculated from patients of the cohort B using the *GLI1*/*IFT20* signature. Patients without a primary cilium were *GLI1-*/*IFT20-*, *GLI1+*/*IFT20-* or *GLI1-*/*IFT20+*. Patients with primary cilia were *GLI1+*/*IFT20+*. Statistical significance (p-value) is indicated. The median survival is also indicated. **B**, Overall survival for the primary cilium signature was calculated from patients of the cohort C using the *GLI1*/*IFT20* signature. Statistical significance (p-value) is indicated. The median survival is also indicated. **C, D and E**, Tumors from patients with the primary cilium signature and tumors from patients with no primary cilium were compared. The level of VDAC1 (**C**), AEP (**D**) and KI67 (**E**) mRNA was determined byRNAseq for the TCGA cohort. Statistical significance (p value) is indicated. **F**, Overall survival of HIF-2 (Oct-4 mRNA expression) or HIF-1/HIF-2 (CA-9 and Oct-4 mRNA expression) patients was calculated from patients of the TCGA Cohort C using the signature. Statistical significance (p-value) is indicated. The median survival is also indicated.

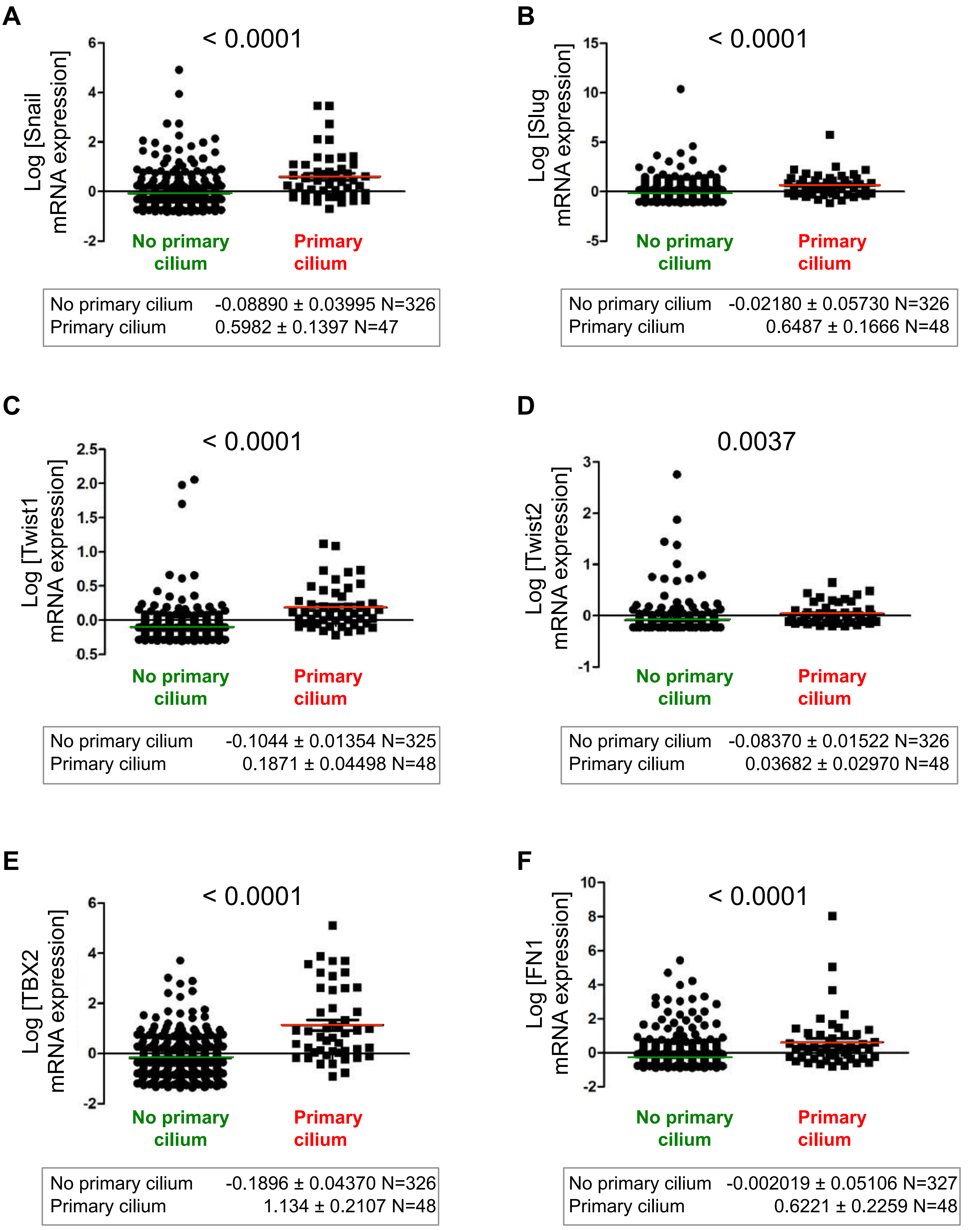

Figure S8. EMT signature. A-F, The level of Snail (**A**), Slug (**B**), Twist1 (**C**), Twist2 (**D**), TBX2 (**E**) and FN1 (**E**) mRNA was determined by RNAseq for the TCGA cohort. Statistical significance (p value) is indicated using the T Test.

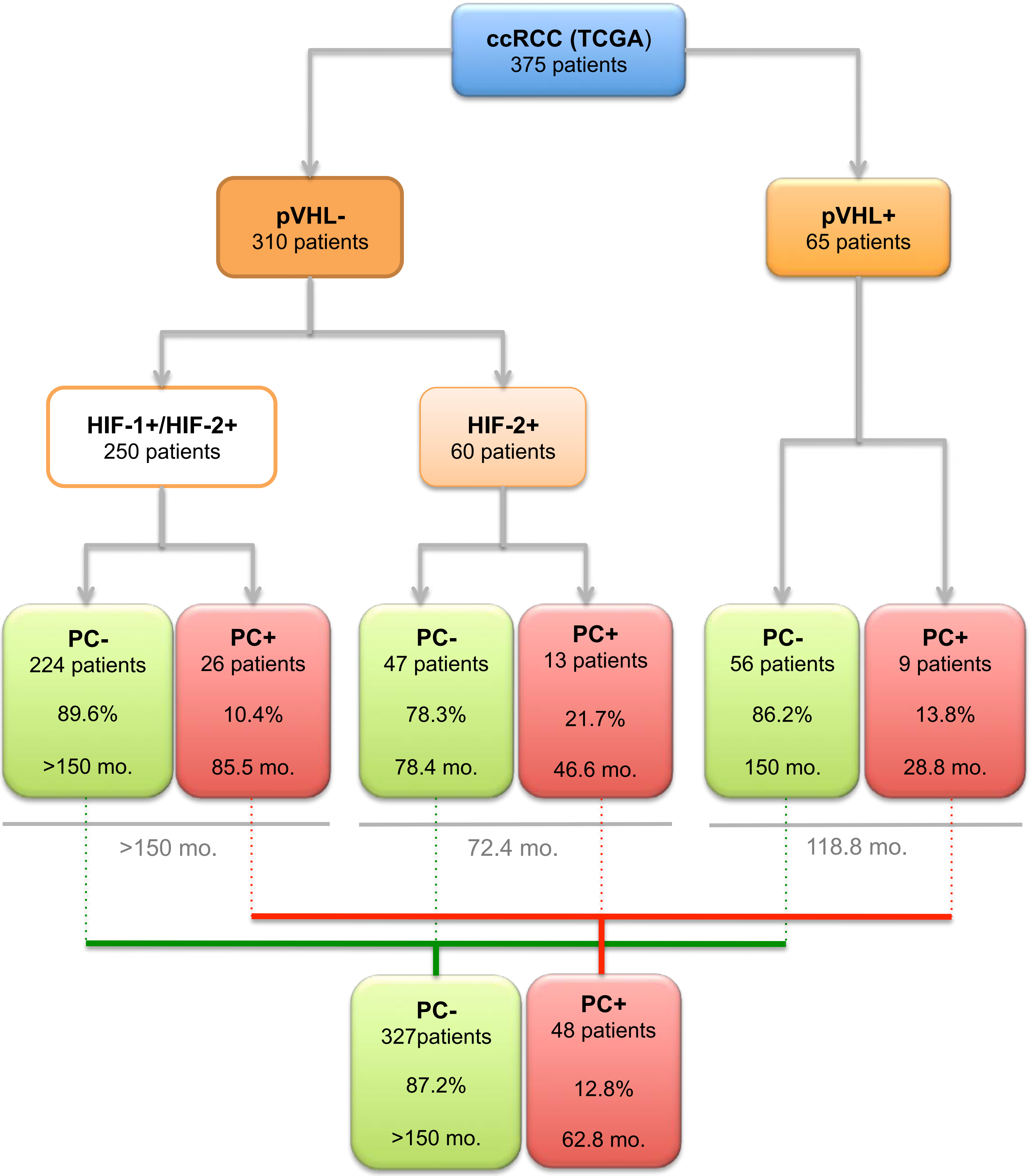

Figure S9. Flow chart showing classification of the 375 patients from the TCGA ccRCC cohort according to the pVHL, HIF and the primary cilium status. Bioinformatic analysis of the TCGA database of 375 ccRCC patients was used to identify groups without (PC-) and with (PC+) primary cilia depending on the pVHL mutation (mutated pVHL, pVHL-; wild type pVHL, pVHL+), the HIF-1 and -2 status and the primary cilium signature. The median survival is indicated in months (mo.).

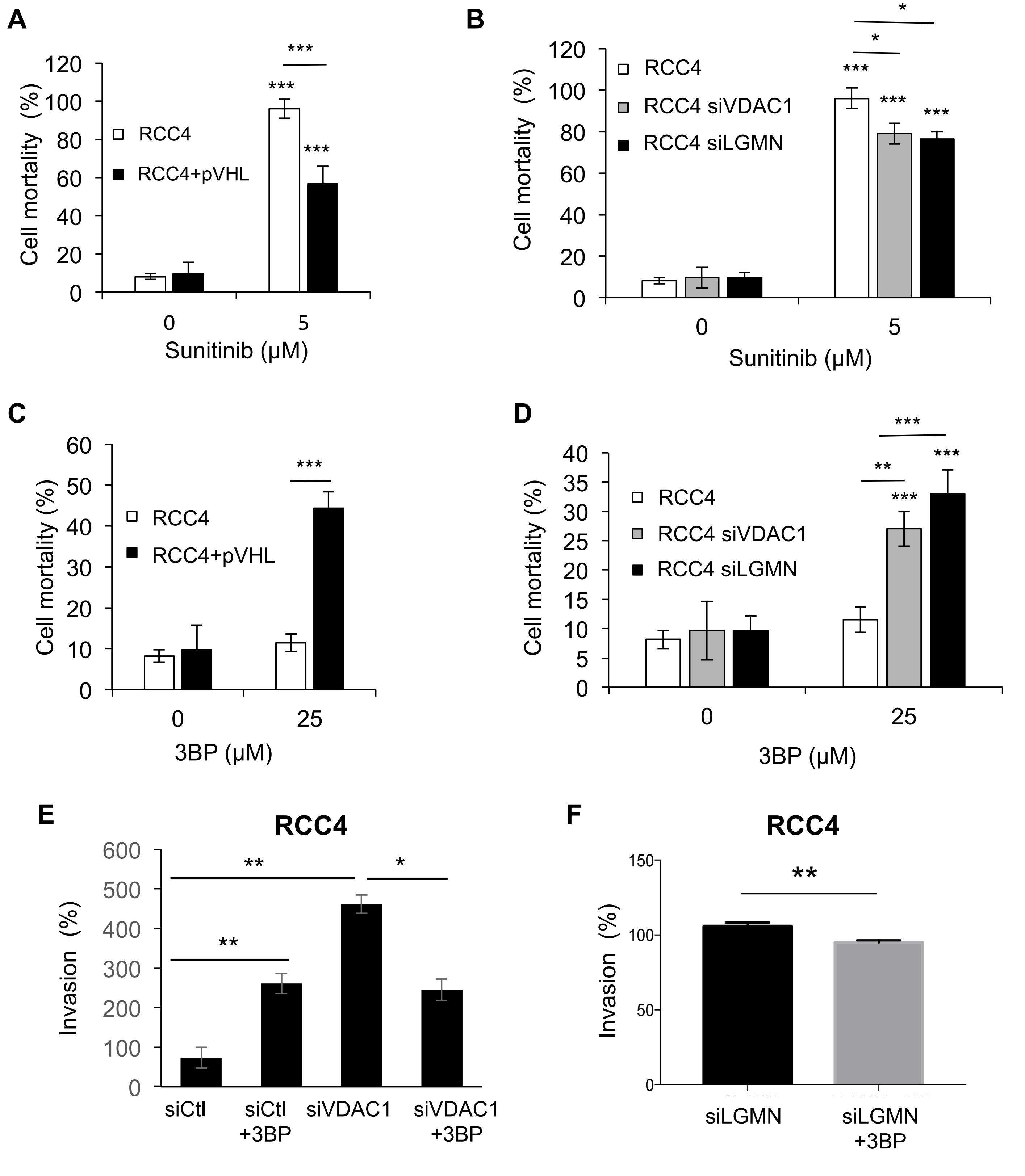

Figure S10. **Absence of VDAC1-DC promotes aggressiveness in RCC4 but allows response to anti-glycolysis treatments. A**, RCC4 and RCC4+pVHL cell lines were incubated in Nx for 72h in the absence or presence of Sunitinib (5µM). **B**, RCC4 cells were transfected with control siRNA (siCtl), siVDAC1 and si*AEP* (40nM). RCC4 cell lines were incubated in Nx for 72h in the absence or presence of sunitinib (5µM). Cell mortality was measured using an ADAM cell counter. **C**, RCC4 and RCC4+pVHL cell lines were incubated in Nx for 72h in the absence or presence of 3BP (25µM). Cell mortality was measured using an ADAM cell counter. **D**, RCC4 cells were transfected with control siRNA (siCtl), siVDAC1 and si*AEP* (40nM). RCC4 cell lines were incubated in Nx for 72h in the absence or presence of 3BP (25µM). Cell mortality was measured using an ADAM cell counter. **E**, Graphic representation of the Boyden chamber cell-based invasion assay using RCC4 cells transfected with siRNA VDAC1 (40nm) compared to Ctl (siCtl) incubated int he absence or presence of 3BP (10µM) for 72h. Invasion assay was performed. * $p<0.05$, ** $p<0.005$, *** $p<0.0005$.

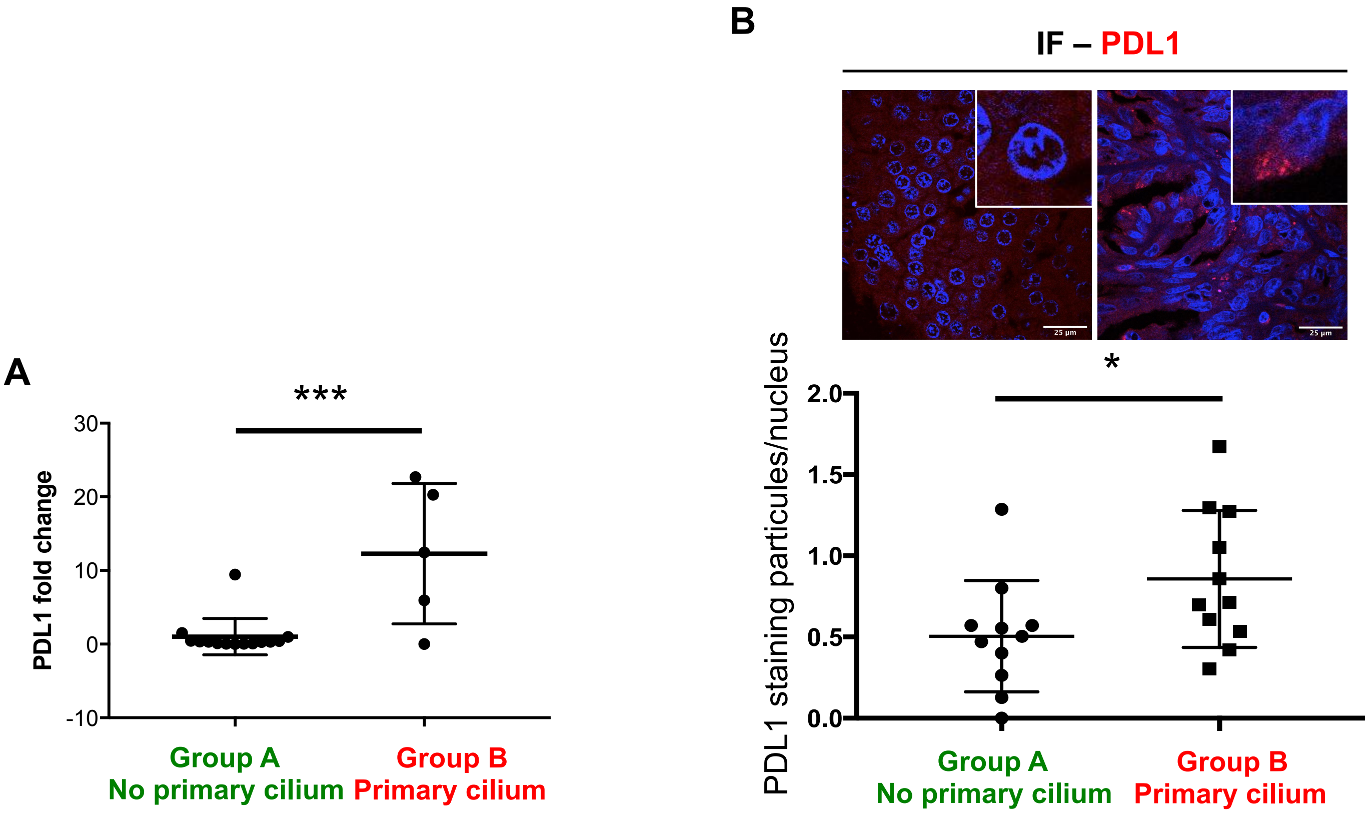

Figure S11. PDL1 is present in group B. A, Graphic representation of PDL1 mRNA expression in patients from Group A compared to patients from Group B. **B,** Representative image of immunofluorescence analysis of patient#8 (Left - Group A) and patient#13 (Right - Group B) studied to evaluate a prediction model of the absence or presence of PDL1 expression. A * p<0.05 and *** p<0.0005 show significant differences.

	Chirurgical date	Age	Sex	TNM	Grade
Patient #1	2017	59	F	pT1A NX MX R0	3
Patient #2	2018	66	M	pT1bNXMX R0	3
Patient #3		60	M	pT1b Nx Mx R0	3
Patient #4		63	M	pT1aNxMxR0	2
Patient #5	2017	71	M	pT1bNxMxR0	2
Patient #6	2017	52	F	T1a Nx Mx R0	NA
Patient #7		56	M	pT2a Nx Mx R0	3
Patient #8		77	M	pT3a N0 Mx R0	3
Patient #9		89	F	pT1a N0 Mx R0	3
Patient #10	2017	78	M	NA	NA
Patient #11	2017	38	M	NA	NA
Patient #12	2018	57	M	pT1b NxMxR0	NA
Patient #13	2018	72	F	pT3a Nx Mx R0	4
Patient #14		58	M	pT3a N0 Mx R0	4
Patient #15	2018	51	M	pT1a	3
Patient #16	2018	53	M	pT3a N0 M1	3
Patient #17	2018	79	M	pT1B Nx Mx R0	2
Patient #18	2017	NA	NA	NA	NA
Patient #19		42	F	pT1a Nx Mx R1	2

Table S1. Summary of clinocopathological parameters of nineteen ccRCC patients from the Pathology Department of Nice (CHU).

	Hospital	Size (cm)	Fuhrman's grade	Stage	Sex	Age	Histology	Fuhrman's grade	Stage	*VHL* status
Patient #1	Bicêtre	6.5	1	1	M	24	VHL-ccRCC	Low	Low	mutated
Patient #2	Bicêtre	2.5	2	2	M	28	VHL-ccRCC	Low	Low	mutated
Patient #3	Necker	1.5	2	2	M	26	VHL-ccRCC	Low	Low	mutated
Patient #4	Necker	4.5	2	1	F	45	VHL-ccRCC	Low	Low	mutated
Patient #5	Bicêtre	4	2	1	M	34	VHL-ccRCC	Low	Low	mutated
Patient #6	Necker	3.5	2	1	F	40	VHL-ccRCC	Low	Low	mutated
Patient #7	Necker	2.3	2	1	M	27	VHL-ccRCC	Low	Low	mutated
Patient #8	Necker	1.3	2	1	M	23	VHL-ccRCC	Low	Low	mutated
Patient #9	Necker	3.5	2	3b	F	40	VHL-ccRCC	Low	High	mutated
Patient #10	Bicêtre	2.5	3	4	M	65	VHL-ccRCC	High	High	mutated
Patient #11	Necker	8.5	3	3a	M	31	VHL-ccRCC	High	High	mutated
Patient #12	Bicêtre	17	4	3a	M	35	VHL-ccRCC	High	High	mutated
Patient #13	St Joseph	6	2	3b	F	75	sporadic ccRCC	Low	High	**wild-type**
Patient #14	Necker	5.5	3	2	F	45	sporadic ccRCC	High	Low	**wild-type**
Patient #15	St Joseph	12	3	2b	M	61	sporadic ccRCC	High	Low	**wild-type**
Patient #16	St Joseph	6	3	3a	M	56	sporadic ccRCC	High	High	**wild-type**
Patient #17	St Joseph	12	3	3b	M	71	sporadic ccRCC	High	High	**wild-type**
Patient #18	St Joseph	11	4	3	M	59	sporadic ccRCC	High	High	**wild-type**
Patient #19	St Joseph	5	1	1b	F	77	sporadic ccRCC	Low	Low	mutated
Patient #20	St Joseph	5	2	1b	M	60	sporadic ccRCC	Low	Low	mutated
Patient #21	St Joseph	12	2	3a	M	85	sporadic ccRCC	Low	High	mutated
Patient #22	St Joseph	5	2	3a	M	76	sporadic ccRCC	Low	High	mutated
Patient #23	St Joseph	1.7	2	1a	M	53	sporadic ccRCC	Low	Low	mutated
Patient #24	St Joseph	6	2	1b	F	70	sporadic ccRCC	Low	Low	mutated
Patient #25	St Joseph	5.5	2	3b	F	77	sporadic ccRCC	Low	High	mutated
Patient #26	St Joseph	5	2	2a	M	83	sporadic ccRCC	Low	Low	mutated
Patient #27	St Joseph	2.5	2	1a	M	69	sporadic ccRCC	Low	Low	mutated
Patient #28	St Joseph	9	3	3b	F	70	sporadic ccRCC	High	High	mutated
Patient #29	St Joseph	7	3	3	F	78	sporadic ccRCC	High	High	mutated
Patient #30	St Joseph	5.5	3	2	M	47	sporadic ccRCC	High	Low	mutated
Patient #31	St Joseph	10	4	3b	F	45	sporadic ccRCC	High	High	mutated
Patient #32	St Joseph	6	4	3b	F	65	sporadic ccRCC	High	High	mutated
Patient #33	St Joseph	10	4	4	M	67	sporadic ccRCC	High	High	mutated
Patient #34	St Joseph	4	2	1b	F	69	sporadic ccRCC	Low	Low	mutated

Table S2. Summary of clinocopathological parameters of thirty-four patients from Cohort A (PREDIR).

Variables	Cohort B (n=43)
**Mean age**	63
**Sex**	
Male	27 (62.8%)
Female	16 (37.2%)
**Furhman grade**	
II	20 (46.5%)
III	16 (37.2%)
IV	7 (16.3%)
**Metastatic status**	
M0	43 (100%)
M1	0 (0%)
**T (size) status**	
T1/2	27 (62.8%)
T3/4	16 (37.2%)
**N status**	
N0	41 (95.3%)
N1	2 (4.7%)
**OS (Months)**	Sup 150

Table S3. Summary of clinocopathological parameters of forty-three patients from Cohort B (Rennes).

p-value=1.5243E-8

Pathologic stage	PC -	PC +
Stage 1/2	237 (74.8%)	16 (34%)
Stage 3/4	80 (25.2%)	31 (66%)

Table S4. Distribution of ccRCC patients from the TCGA database depending on the primary cilium (PC) signature and pathologic stage. p-value between No primary cilium (PC-) and Primary cilium (PC+) is indicated. For 11 patients, the pathologic stage is missing in the database.

Patients	Time of treatment (months)	Progression	Time after treatment	Death	Best response (cycle 2)	T (TNM)	N (TNM)	M (TNM)	Age	Sexe	Statut IFT20	Statut Gli1
#1	20.0	0	45.5	0	Partial response	1	0	1	56	MALE	-	+
#2	8.1	1	22.0	1	Progression	3	1	1	66	MALE	+	+
#3	8.3 (toxicity)	0	49.9	0	Stable	1	X	1	74	MALE	-	+
#4	25.8	1	48.6	1	Stable	2	X	1	61	FEMALE	-	+
#5	17.5	1	29.7	1	Stable	3	X	1	61	MALE	+	-
#6	21.2	0	21.2	0	Complete response	1	X	1	55	MALE	+/-	-

Table S5. Characteristic of patients treated with sunitinib.
